# The canonical α-SNAP is essential for gametophytic development in Arabidopsis

**DOI:** 10.1371/journal.pgen.1009505

**Published:** 2021-04-22

**Authors:** Fei Liu, Ji-Peng Li, Lu-Shen Li, Qi Liu, Shan-Wei Li, Ming-Lei Song, Sha Li, Yan Zhang

**Affiliations:** 1 Department of Plant Biology and Ecology, College of Life Sciences, Nankai University, Tianjin, China; 2 State Key laboratory of Crop Biology, College of Life Sciences, Shandong Agricultural University, Tai’an, China; Peking University, CHINA

## Abstract

The development of male and female gametophytes is a pre-requisite for successful reproduction of angiosperms. Factors mediating vesicular trafficking are among the key regulators controlling gametophytic development. Fusion between vesicles and target membranes requires the assembly of a fusogenic soluble *N*-ethylmaleimide sensitive factor attachment protein receptors (SNAREs) complex, whose disassembly in turn ensures the recycle of individual SNARE components. The disassembly of post-fusion SNARE complexes is controlled by the AAA^+^ ATPase *N*-ethylmaleimide-sensitive factor (Sec18/NSF) and soluble NSF attachment protein (Sec17/α-SNAP) in yeast and metazoans. Although non-canonical α-SNAPs have been functionally characterized in soybeans, the biological function of canonical α-SNAPs has yet to be demonstrated in plants. We report here that the canonical α-SNAP in Arabidopsis is essential for male and female gametophytic development. Functional loss of the canonical α-SNAP in Arabidopsis results in gametophytic lethality by arresting the first mitosis during gametogenesis. We further show that Arabidopsis *α-SNAP* encodes two isoforms due to alternative splicing. Both isoforms interact with the Arabidopsis homolog of NSF whereas have distinct subcellular localizations. The presence of similar alternative splicing of human *α-SNAP* indicates that functional distinction of two α-SNAP isoforms is evolutionarily conserved.

## Author summary

Vesicular trafficking among different endomembrane compartments ensures membrane homeostasis, protein targeting, and finally the survival of eukaryotic cells. Fusion between vesicle and target membranes is mediated by tetrameric SNARE complexes, whose disassembly ensures the recycle of individual SNARE components. The disassembly of post-fusion SNARE complexes is controlled by NSF/Sec18 and α-SNAP/Sec17 both in yeast and metazoans. Recent reports show that a naturally occurring, truncated α-SNAP allele suppresses parasitic nematode infection in soybeans. However, a role of the canonical α-SNAP has yet to be demonstrated in plants. We report here that the canonical α-SNAP in Arabidopsis is essential for fertility. Mutations in *α-SNAP* arrest mitosis and causes gametophytic lethality. In addition, we demonstrated that Arabidopsis *α-SNAP* encodes two functional isoforms. Interesting, the single *α-SNAP* gene in human also encodes two similarly distinct isoforms. Thus, the study of Arabidopsis *α-SNAP* provides important hints to that of human *α-SNAP*.

## Introduction

The development of male and female gametophytes is a pre-requisite for successful reproduction of angiosperms. In angiosperms, megagametogenesis [1] and microgametogenesis [2, 3] produce female and male gametophytes, respectively. During megagametogenesis, meiosis of a megaspore mother cell produces four megaspores, among which only one survives as functional megaspore (FM). FM undergoes three rounds of mitosis and cellularization to develop into an embryo sac, i.e. the female gametophyte [1]. During microgametogenesis, meiosis of a microspore mother cell gives rise to a tetrad of microspores. After being released from the tetrad, each microspore goes through an asymmetric cell division, referred to as pollen mitosis I (PMI), to produce a bicellular microspore containing a generative cell and a vegetative nucleus. The generative cell then undergoes another mitotic event, called pollen mitosis II (PMII), to produce two sperm cells enclosed in pollen together with the vegetative nucleus [2, 3].

Regulators of mitosis [4–7], of ribosomal biogenesis [8–12], and of endomembrane integrity [13–19] are major factors controlling gametogenesis. Soluble *N*-ethylmaleimide sensitive factor attachment protein receptors (SNAREs) are coiled-coil domain proteins regulating vesicular fusion [20, 21] between two membranous compartments, often vesicles and organelles within the endomembrane system. A fusogenic SNARE complex consists of four SNARE proteins [20, 21]. Mutations of SNAREs or their interacting partners often compromise gametophytic transmission [17–19, 22]. Indeed, functional loss of Arabidopsis YKT61, an R-SNARE protein, resulted in complete male and female gametophytic lethality [23], suggesting that SNARE-mediated membrane fusion is essential for gametophytic viability.

Vesicle-target membrane fusion not only depends on the assembly of tetrameric SNARE complex but also its disassembly so that the components of post-fusion SNARE complexes can be recycled [24]. Studies in yeast and metazoans demonstrated that the disassembly of post-fusion SNARE complexes is controlled by the AAA^+^ ATPase *N*-ethylmaleimide-sensitive factor (NSF/Sec18) and soluble NSF attachment protein (α-SNAP/Sec17), which perform ATP-dependent disassembly of *cis*-SNARE complexes, liberating SNAREs for subsequent assembly of *trans*-complexes for fusion [25, 26]. In addition to being the partner for NSF, α-SNAP performs a regulatory role in SNARE disassembly [24] or moonlights in other cellular processes [27, 28].

Recent studies in soybean showed that a naturally occurring, truncated α-SNAP allele, i.e. non-canonical α-SNAP, suppresses parasitic nematode infection [29–31]. The non-canonical α-SNAP may be derived from neofunctionalization after genome duplication [31, 32]. The non-canonical α-SNAP did not interact with the NSF homolog in soybean and its enhanced expression depleted the abundance of SNARE-recycling 20S complexes [29, 30]. Naturally occurring, truncated alleles of α-SNAP confer resistance against nematodes in soybean while the expression of a canonical α-SNAP counteracted the cytotoxicity of resistance-type Rhg1 α-SNAP [31, 32]. These results suggested that the non-canonical α-SNAP interferes with the role of the canonical α-SNAP in SNARE disassembly. However, the biological function of canonical α-SNAPs have yet to be demonstrated in plants.

We report here that the canonical *α-SNAP* in the Arabidopsis genome, designated *ASNAP*, is essential for male and female gametophytic development. By CRISPR/Cas9-mediated genomic editing, we generated and characterized *asnap* mutants. Functional loss of *ASNAP* resulted in gametophytic lethality such that both male and female gametophytes could not be transmitted. Specifically, functional loss of *ASNAP* caused mitotic arrest of unicellular microspores and of functional megaspores (FM), suggesting that *ASNAP* is essential for mitotic cell cycle progression during gametophytic development. We show that Arabidopsis *ASNAP* encodes two isoforms due to alternative splicing, both of which interact with the Arabidopsis NSF. The presence of similar alternative splicing of human *α-SNAP* indicates that functional distinction of two α-SNAP isoforms is evolutionarily conserved.

## Results

### Arabidopsis encodes one canonical α-SNAP

**Fig 1:**
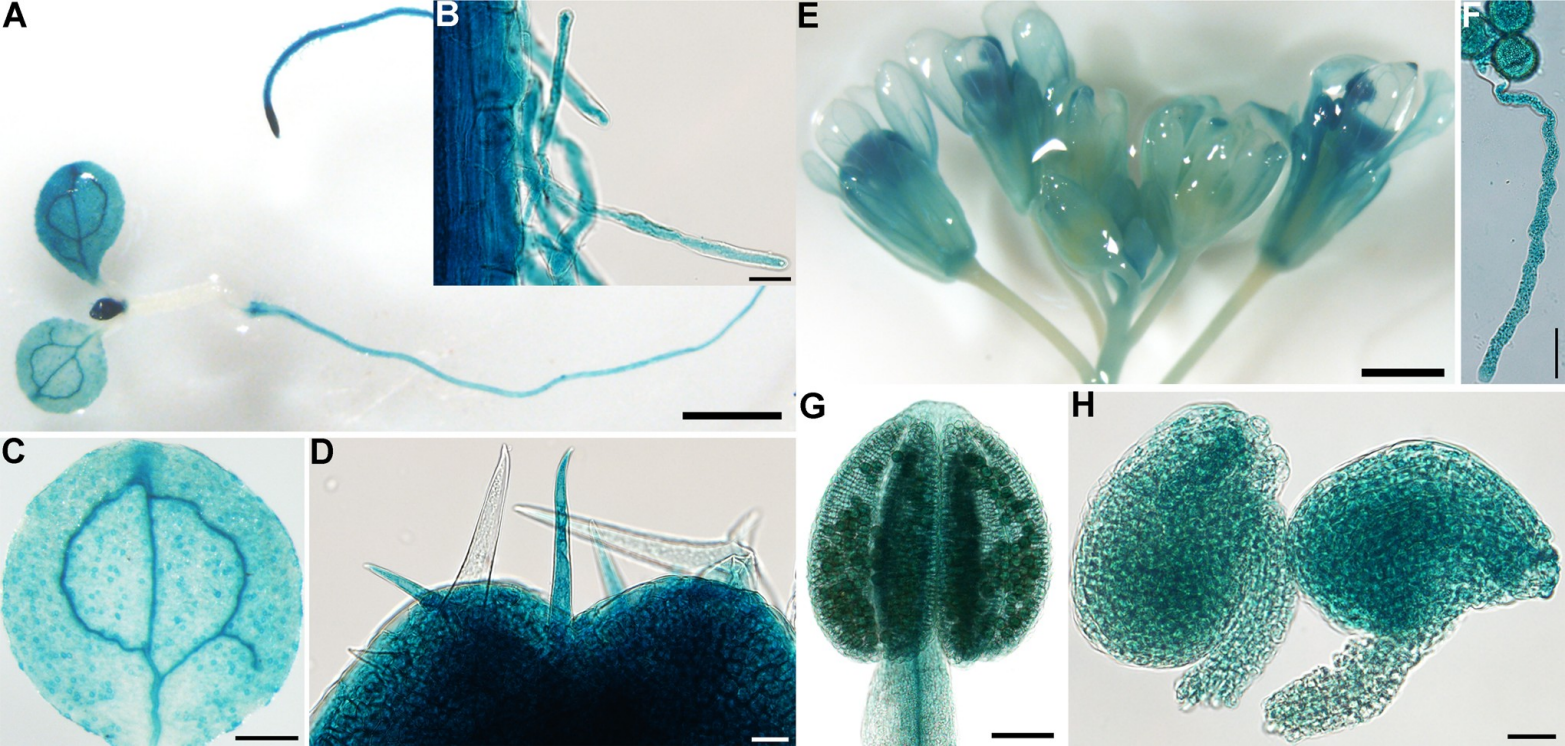
Arabidopsis *ASNAP* is constitutively expressed. (A-H) Representative histochemical GUS staining of a seedling (A), root hairs (B), a cotyledon (C), trichomes on initiating leaves (D), an inflorescence (E), pollen grains and a pollen tube (F), a mature anther (G), and mature ovules (H) from ASNAPg:GUS transgenic plants. Bars = 1 mm for (A, E), 20 μm for (B, D, F, H), 200 μm for (C), 100 μm for (G).

By sequence alignment, one canonical *α-SNAP* whose protein products contain N-terminal, central, and C-terminal domains similar to animal α-SNAP homologs are encoded in the Arabidopsis genome. To determine its expression pattern, we generated ASNAPg:GUS transgenic plants expressing genomic-GUS translational fusion of *ASNAP*. By histochemical GUS staining, we detected GUS signals in various tissues and developmental stages, including seedlings, leaves, roots, reproductive organs, trichomes, root hairs as well as pollen tubes (Fig 1). The constitutive expression of *ASNAP* is consistent with its role as a canonical α-SNAP.

### Generation and characterization of *asnap* mutants

**Fig 2:**
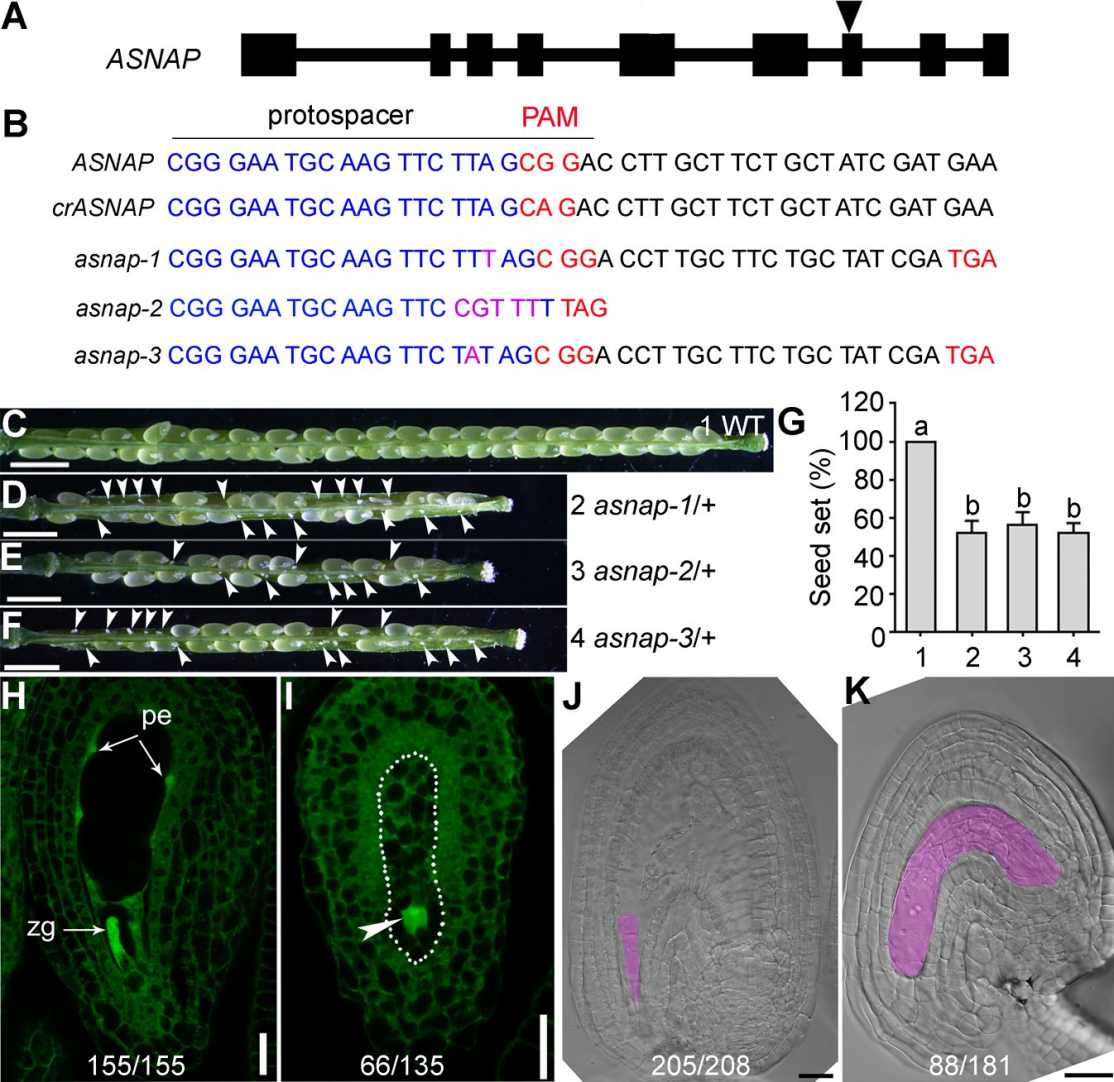
Functional loss of *ASNAP* causes reduced fertility. (A) The *ASNAP* genomic structure. Inverted triangle indicates Cas9-target site. (B) Generation of the *asnap* mutants and the Cas9-resistant *ASNAP* (*crASNAP*). Dark blue indicates the protospacer sequence; red indicates the PAM sequence and pre-stop codon generated during genomic editing; pink indicates nucleotide insertions generated by CRISPR/Cas9 in the corresponding *asnap* mutants. Pre-stop codons were generated at the 730–732 (*asnap-1* and *asnap-3*) or 709–711 bp (*asnap-2*) of the *ASNAP* CDS. (C-G) Representative seed set of wild type, *asnap-1*/+, *asnap-2*/+, and *asnap-3*/+. (G) Quantification of seed sets. Genotypes are indicated in (C-F). Results are means ± SD (n>10). Different letters indicate significantly different groups (One-Way ANOVA, Tukey’s multiple comparisons test, P<0.05). (H-I) Representative CLSM of an ovule from wild type (H) or *asnap-1*/+ (I) pollinated with wild-type pollen at 24 hours after pollination (HAP). Dotted line illustrate the embryo sac; arrowhead points to a single nucleus in the embryo sac; zg indicates the elongating zygote; pe indicates peripheral endosperm. Numbers at the bottom indicate displayed/total examined ovules. (J-K) Representative differential interference contrast (DIC) image of an ovule from wild type (J) or *asnap-1*/+ (K) pollinated with wild-type pollen at 24 HAP. Pink highlights the developing embryo in (J) or the embryo sac in (K). Numbers at the bottom indicate displayed/total examined ovules. Bars = 1 mm for (C-F); 20 μm for (H-K).

Because no valid T-DNA insertion lines were available for *ASNAP* from all stock centers, we used the genome-editing technology CRISPR/Cas9 [33, 34] to generate *asnap* mutants. We transformed Cas9-ASNAP driven by an egg cell-specific promoter [35] and screened its transformants for the editing of the *ASNAP* genomic locus. We identified three allelic mutations of *ASNAP*, in which nucleotide insertions resulted in pre-mature stop codons in the coding sequence of *ASNAP* (Fig 2A and 2B). Because *asnap-1* is an allele repeatedly obtained, most experiments including the complementation assays were performed with *asnap-1*/+.

All three *asnap* mutant alleles were only obtained in their heterozygous forms, i.e. *asnap-1*/+, *asnap-2*/+, and *asnap-3*/+. Silique analysis showed that around 50% ovules were tiny white and wrinkled in the self-fertilized *asnap*/+ plants (Fig 2C–2G), indicating reduced fertility. To determine what have caused the seed set reduction, we examined *asnap-1*/+ pistils pollinated with wild-type pollen at 24 hours after pollination (HAP) by confocal laser scanning microscopy (CLSM) and whole-mount ovule clearing. At 24 HAP, wild-type ovules contained elongating zygotes or early embryos and peripheral endosperms (Fig 2H and 2J), indicating the completion of fertilization. By contrast, half of the *asnap-1*/+ ovules contained a single nucleus (Fig 2I) with no detectable peripheral endosperms or embryos (Fig 2I and 2K). These results suggested that the 50% white and wrinkled ovules in the heterozygous *asnap*/+ plants were not fertilized. Reciprocal crosses and seed set assays between *asnap-1*/+ and wild type showed that the reduced seed sets of *asnap-1*/+ were due to female gametophytic defects (S1 Fig) and *asnap-1* was not transmitted either through the male or the female (Table 1), suggesting gametophytic lethality.

**Table 1:** *ASNAP* is essential for both male and female transmission.

Progeny	Genotype ^a^				
	*ASNAP*	*asnap-1*/+	*asnap-1*	Ratio	Expected Ratio
♀*asnap-1*/+ × ♂WT	82	6 ^a^	NA	1:0.07 ^b^	1:1
♀WT × ♂*asnap-1*/+	72	0	NA	1:0 ^b^	1:1
♀*asnap-1*/+ × ♂*asnap-1*/+	84	0	0	1:0:0 ^c^	1:2:1

^a^ Re-knockout by Cas9-ASNAP at the next generation.

^b^ Significantly different from the segregation ratio 1:1 (χ2 < χ20.05,2).

^c^ Significantly different from the Mendelian segregation ratio (χ2 < χ20.05,1).

### Pollen development is defective in *asnap*

**Fig 3:**
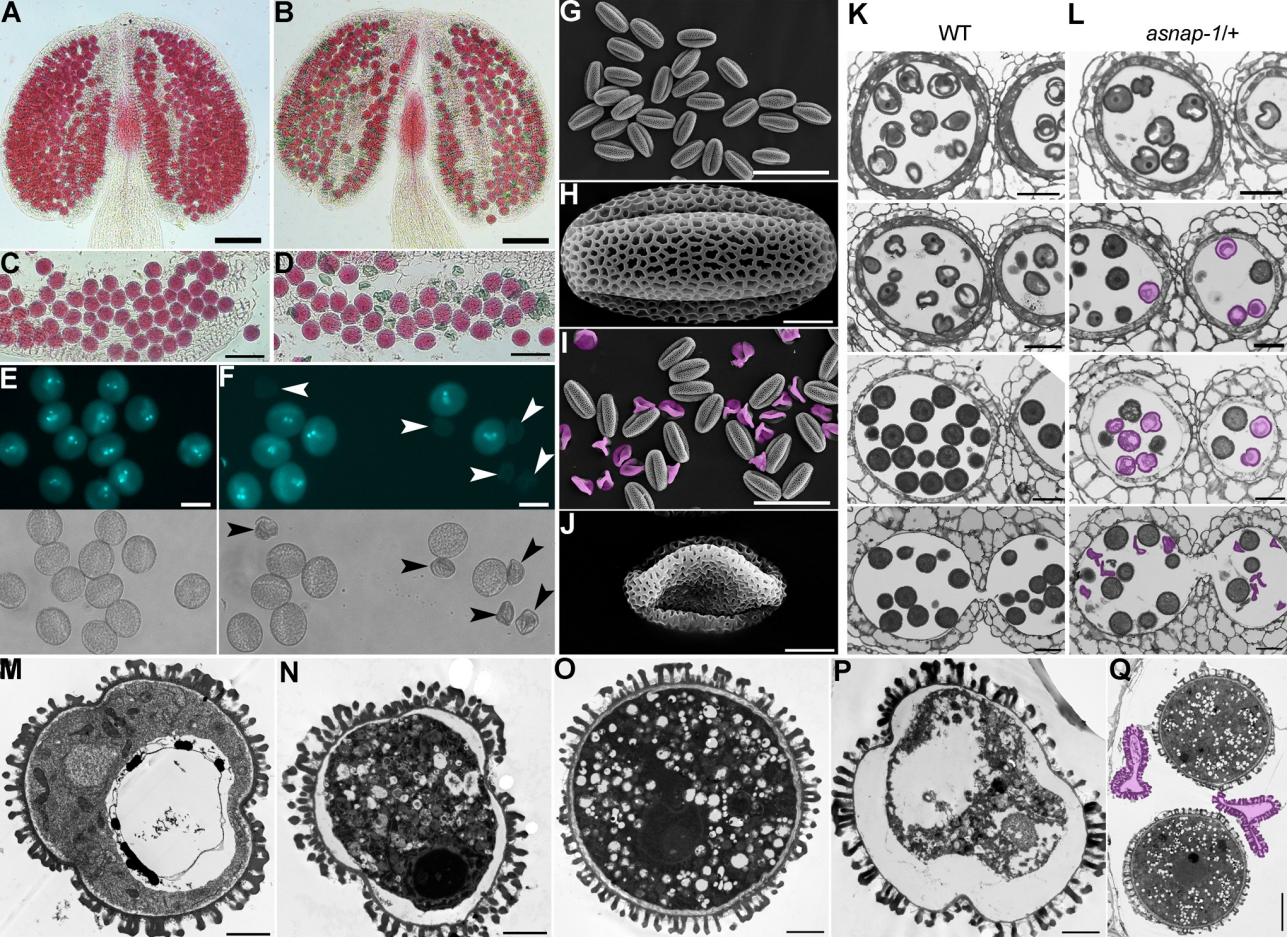
*ASNAP* is essential for male gametophytic development. (A-D) Alexander staining of a representative anther (A, B) or mature pollen grains (C, D) from wild type (A, C) or *asnap-1*/+ (B, D). (E-F) DAPI staining of mature pollen grains from wild type (E) or *asnap-1*/+ (F). Bright-field images are shown at the bottom of corresponding fluorescent images. Arrowheads point at aborted pollen grains. (G-J) Scanning electron micrographs (SEMs) of mature pollen from wild type (G, H) or *asnap-1*/+ (I, J). Aborted pollen grains are pseudo-colored in pink. (K-L) Representative semi-thin transverse sections of developing wild-type (K) or *asnap-1*/+ (L) anthers. From top to bottom: at stage 9, stage 10, stage 11, or stage 12. Defective microspores are pseudo-colored in pink. (M-Q) Transmission electron micrographs (TEMs) of microspores in *asnap-1*/+ at stage 10 (M, N), stage 11 (O, P), or stage 12 (Q). Wild-type-like microspores are shown in (M, O) while defective microspores are shown in (N, P). Two normally developed tricellular microspores are shown together with two aborted ones (pseudo-colored) in (Q). Bars = 100 μm for (A, B); 50 μm for (C, D, G, I); 20 μm for (E, F, K, L); 5 μm for (H, J); 2 μm for (M-P); 10 μm for (Q).

To determine the cause for complete zero male transmission, we examined pollen development of *asnap-1*/+ mutants by Alexander staining for pollen cytoplasmic viability (Fig 3A–3D), by DAPI staining for the development of tricellular pollen (Fig 3E and 3F), and by scanning electron micrographs (SEMs) for pollen morphology (Fig 3G–3J). Half of the pollen grains from *asnap-1*/+ were aborted either by Alexander staining, by DAPI staining, or by SEM (Figs 3 and S2), indicating that functional loss of *ASNAP* caused pollen abortion. To determine exactly what occurred during pollen development by *ASNAP* loss-of-function, we performed plastic embedding and transverse sectioning (Fig 3K and 3L), as well as ultrastructure studies of anthers at different developmental stages (Fig 3M–3Q). In wild type, microspores in stage 10 anthers are unicellular, containing a large central vacuole with electron-dense materials inside (Fig 3K and 3M); microspores in stage 11 anthers contain a generative cell, a vegetative nucleus, as well as numerous small vacuoles (Fig 3K and 3O). By contrast, in *asnap-1*/+ anthers at stage 10, some of the unicellular microspores showed the detachment of cytoplasmic contents from pollen coat and disrupted organization of intracellular structures (Fig 3L and 3N). In *asnap-1*/+ anthers at stage 11, some microspores did not go through PMI (Fig 3P). Instead, they showed disintegration of internal organization (Fig 3L and 3P). In *asnap-1*/+ anthers at dehiscing stages, half of the microspores were degenerated (Fig 3L and 3Q). The defective pollen development in *asnap-1*/+ was confirmed by CLSM optical sections of developing anthers (S2 Fig). These results suggested that PMI was arrested by *ASNAP* loss-of-function, resulting in complete abortion of *asnap-1* microspores. Consistent with the gametophytic defects, the deposition and organization of pollen coats were unaffected in *asnap-1*/+ (Fig 3I, 3J, 3N,3P and 3Q).

### Defective embryo sacs by *ASNAP* loss-of-function fail to attract pollen tubes

**Fig 4:**
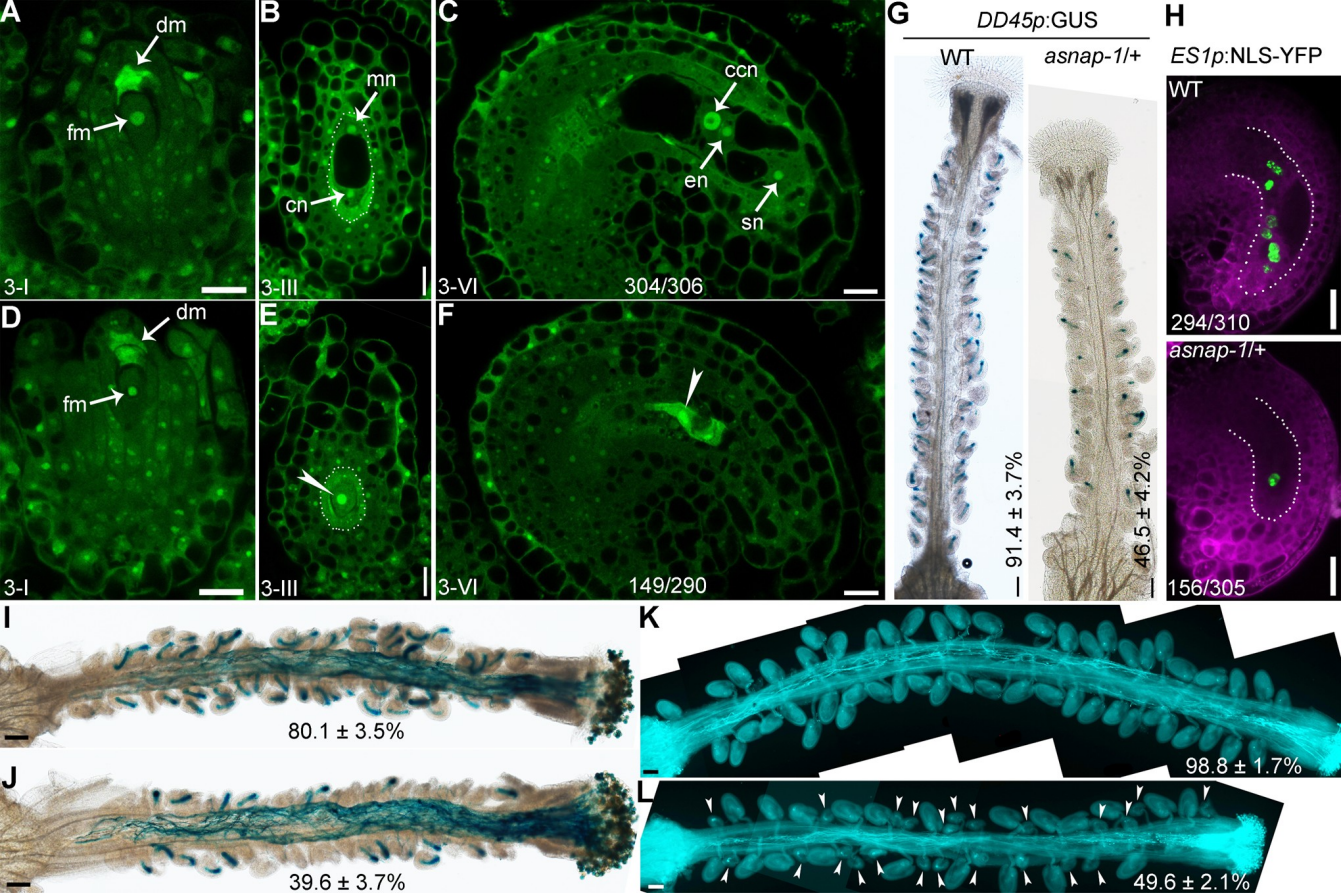
Functional loss of *ASNAP* results in the arrest of female gametogenesis. (A-F) CLSMs of representative wild-type (A-C) or *asnap-1*/+ (D-F) ovules at stage 3-I (A, D), stage 3-III (B, E), or stage 3-VI (C, F). ccn, central cell nucleus; cn, chalazal nucleus; dm, degenerating megaspore; en, egg cell nucleus; fm, functional megaspore; mn, micropyle nucleus; sn, synergid nucleus. Dotted lines in (B, E) illustrate the embryo sacs. Arrowheads point to the single nucleus in embryo sacs. Numbers at the bottom indicate displayed/total examined ovules. (G) Representative histochemical GUS staining of a *DD45p*:GUS (WT) or *DD45p*:GUS*;asnap-1*/+ (*asnap-1*/+) pistil at maturation. The percentage of GUS-positive ovules are means ± SD (n>10). (H) Overlaid CLSM images of a lysotracker red (magenta)-stained *ES1p*:NLS-YFP (WT) or *ES1p*:NLS-YFP;*asnap-1*/+ transgenic ovule (*asnap-1*/+) at stage 3-V. Numbers at the bottom indicate displayed/total examined ovules. (I-J) Histochemical GUS staining of a wild-type (I) or a *asnap-1*/+ pistil (J) pollinated with *LAT52p*:GUS pollen at 12 HAP. Percentage of ovules targeted by pollen tubes is shown at the bottom. Results are means ± SD (n>10). (K-L) Aniline blue staining of a wild-type (K) or a *asnap-1*/+ pistil (L) pollinated with wild-type pollen at 48 HAP. Arrowheads in (L) point at unfertilized ovules. Several overlapping high-magnification images were taken and overlaid to show the whole pistil in (I-L). Percentage of fertilized ovules is shown at the bottom. Results are means ± SD (n>10). Wild type and *asnap-1*/+ are significantly different at both pollen tube attraction and fertilization (*t*-test, P<0.05). Bars = 10 μm for (A-F); 100 μm for (G, I-L); 20 μm for (H).

To determine for the cause of reduced female fertility in *asnap-1*/+, we performed CLSM of ovules at various developmental stages. Optical sections of developing embryo sacs indicated that the formation of FM was not affected in *asnap-1*/+ plants (Fig 4A and 4D). In wild type, a FM goes through three rounds of mitosis and finally develops into a mature embryo sac with a central cell, an egg cell, and two synergid cells (Fig 4C). By contrast, half of the ovules in *asnap-1*/+ pistils had a defective embryo sac containing only one, sometimes two nuclei (Fig 4F) due to the defects of the first mitosis (Fig 4E). To confirm the embryo sac developmental defect, we introduced an egg cell-reporter transgene *DD45p*:*GUS* into *asnap-1*/+. In *DD45p*:*GUS* plants, all mature ovules were positive for GUS signals, indicating the presence of egg cells (Fig 4G). By contrast, only half of the ovules in *DD45p*:*GUS*;*asnap-1*/+ pistils were positive for GUS signals (Fig 4H), indicating the defective embryo sac development of *asnap-1*. Consistently, the transgene *ES1p*:*NLS-YFP* in wild type labeled all 7–8 nuclei of each embryo sac whereas labeled mostly one nucleus in embryo sacs of half ovules of *asnap-1*/+ (Fig 4H). These results demonstrated that functional loss of *ASNAP* caused the arrest of mitosis during female gametophytic development.

To determine whether the defective female gametophytic development by *ASNAP* loss-of-function resulted in female sterility, we pollinated emasculated *asnap-1*/+ pistils with *LAT52p*:*GUS* pollen, which allows histochemical GUS staining and examination of pollen tube growth and guidance *in vivo*. At 12 HAP, histochemical GUS staining of wild-type pistils showed that most ovules were targeted by a pollen tube (Fig 4I). By contrast, less than half *asnap-1*/+ ovules were targeted by a pollen tube at the same stage (Fig 4J). By examining wild-type (Fig 4K) or *asnap-1*/+ pistils (Fig 4L) emasculated and pollinated with wild-type pollen at 48 HAP, we confirmed that half of the *asnap-1*/+ ovules could not be fertilized (Fig 4L). These results demonstrated that defective embryo sac development by *ASNAP* loss-of-function resulted in the failure of pollination and of fertilization.

### Defective gametophytic development of *asnap-1* is mimicked by gametophytic downregulation of *ASNAP* and rescued by genomic *ASNAP*

**Fig 5:**
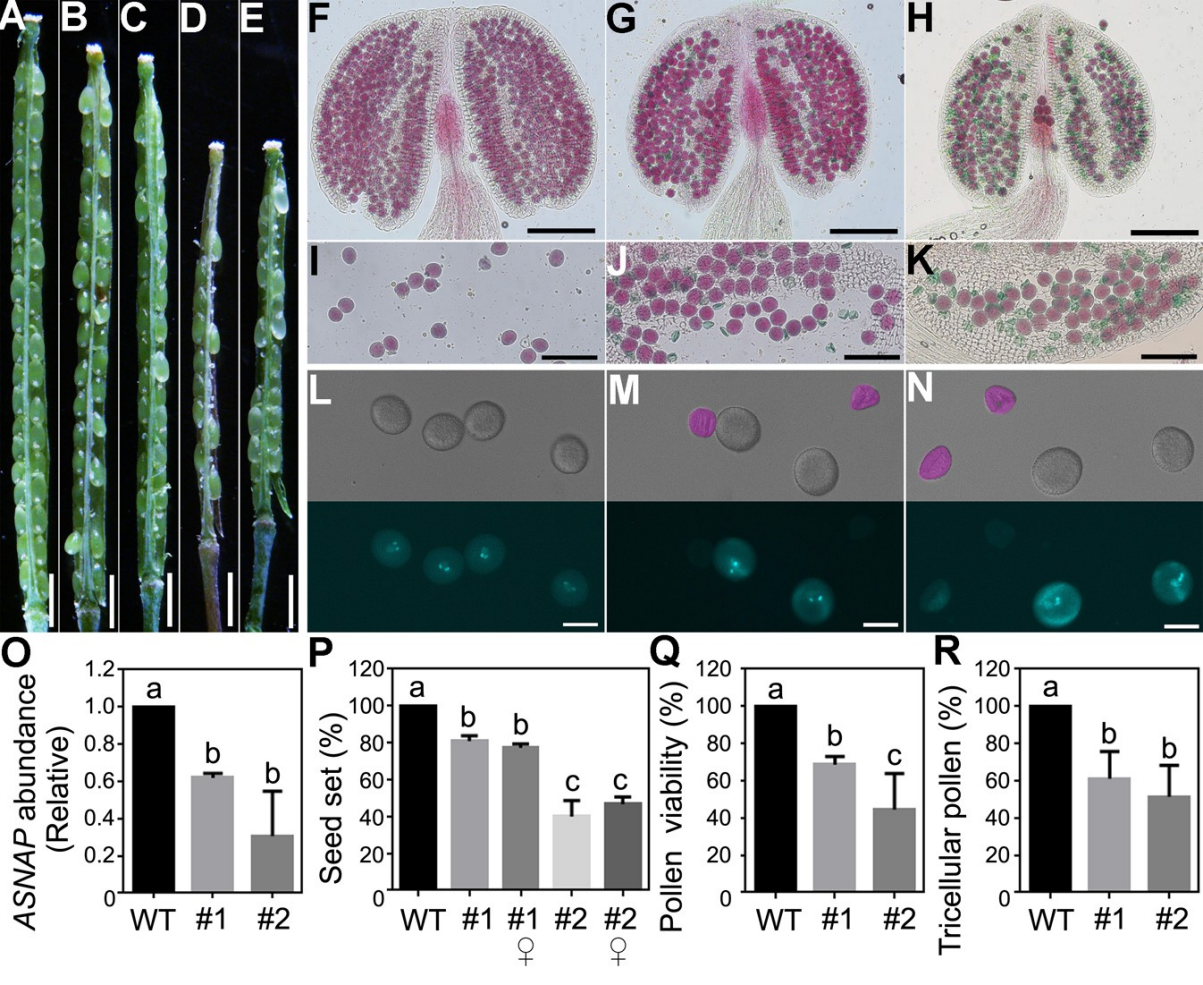
Downregulating *ASNAP* with a gametophyte-specific promoter mimics *ASNAP* loss-of-function. (A-E) Representative silique from the wild type (A), self-fertilized *GPR1p*:amiR-ASNAP#1 (amiR#1) (B), amiR#1 pollinated with wild-type pollen (C), self-fertilized amiR#2 (D), or amiR#2 pollinated with wild-type pollen (E). (F-K) Alexander staining of a dehiscing anther (F-H) or pollen grains (I-K) from wild-type (F, I), amiR#1 (G, J) or amiR#2 (H, K) plants. (L-N) DAPI staining of mature pollen from wild-type (L), amiR#1 (M) or amiR#2 (N) plants. (O) Transcript abundance of *ASNAP* in wild type and two lines (#1 and #2) of the amiR plants. RNAs were extracted from inflorescences. Results are means ± SE (n = 3). Different letters indicate significant different groups (One-Way ANOVA, Tukey’s multiple comparisons test, P<0.05). (P-R) Percentage of seed set (P), of pollen viability by alexander staining (Q), or of tricellular pollen by DAPI staining (R). Results are means ± SD (n>10) for (P). Results are means ± SE (n = 4 involving over 300 pollen grains) for (Q, R). Different letters indicate significantly different groups (One-Way ANOVA, Tukey’s multiple comparisons test, P<0.05). Bars = 1 mm for (A-E); 100 μm for (F-H); 50 μm for (I-K); 20 μm for (L-N).

To provide evidence that *ASNAP* is essential for gametophytic development, we generated an artificial microRNA construct for *ASNAP*, driven by a gametophytic linage promoter *GPR1p* [36]. The expression of *amiR-ASNAP* resulted in reduced seed set (Fig 5A–5E and 5P), defective pollen development (Fig 5F–5N and 5Q–5R), mimicking the male and female gametophytic defects of *ASNAP* loss-of-function. By quantitative real-time PCRs (RT-qPCRs), we confirmed that the *GPR1p*:*amiR-ASNAP* transgene did reduce the expression levels of *ASNAP* (Fig 5Q). These results supported an essential role of *ASNAP* in gametophytic development.

Because *ASNAP* is constitutively expressed (Fig 1), we wondered whether downregulating *ASNAP* in sporophytic tissues could affect plant growth. To this purpose, we generated *UBQ10p*:*amiR-ASNAP* transgenic plants. Transcript analysis verified the downregulation of *ASNAP* in different transgenic lines (S3 Fig). Two lines representing medium or strong downregulation of *ASNAP* were used for further analysis (S3 Fig). Downregulating *ASNAP* compromised plant growth (S3 Fig). Fertility of the *UBQ10p*:*amiR-ASNAP* transgenic plants was significantly reduced (S3 Fig). However, unlike that of *asnap*/+, *UBQ10p*:*amiR-ASNAP* transgenic plants produced pollen with defective pollen coat structure (S3 Fig), indicating sporophytic defects. These results support a role of *ASNAP* in sporophytic tissues in addition to that in gametophytes.

**Fig 6:**
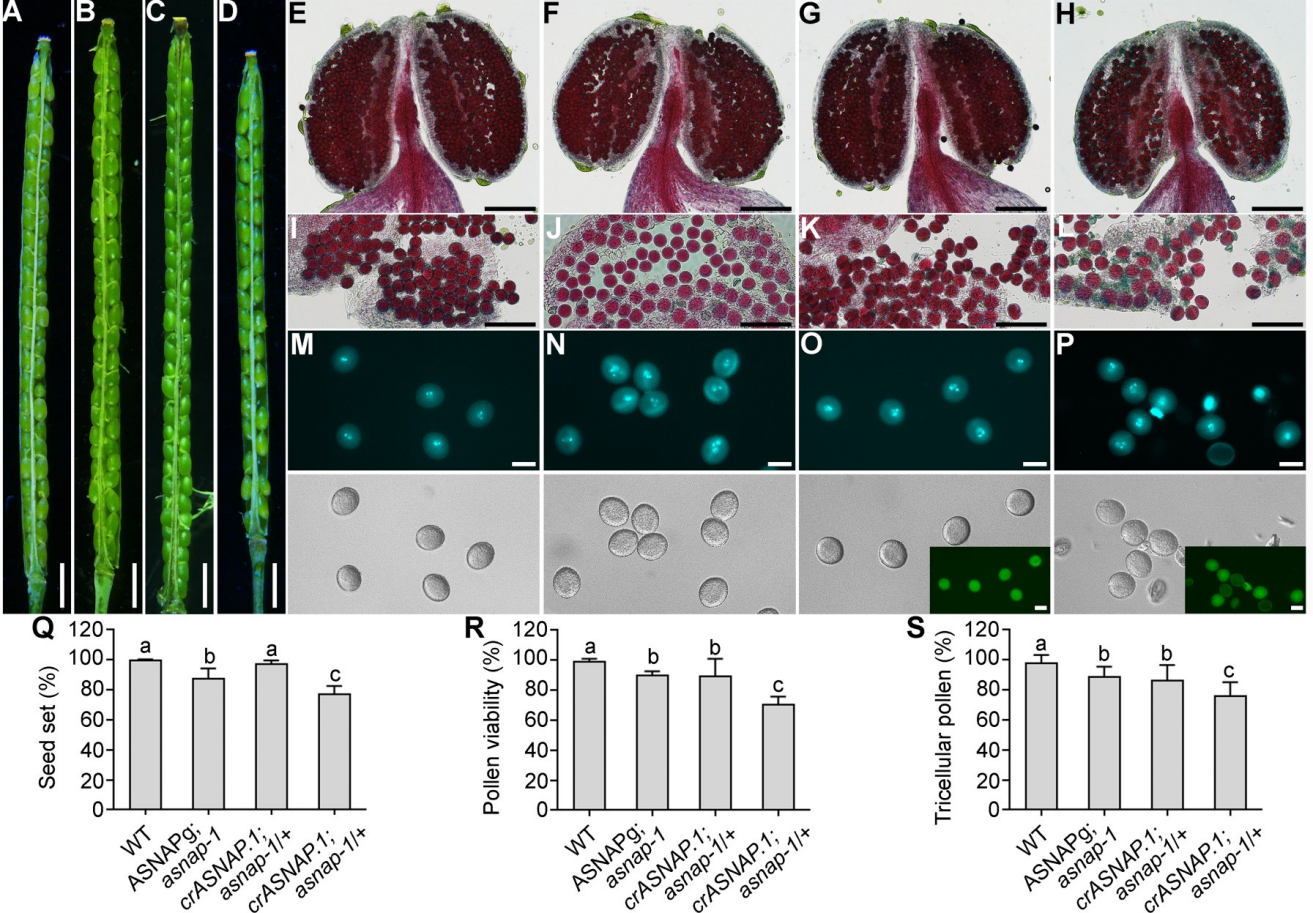
Complementation of *ASNAP* loss-of-function fully by the *ASNAP* genomic fragment whereas partially by each of the two splicing variants. (A-D) Representative silique from the wild type (A), *ASNAPg*;*asnap-1* (B), *UBQ10p*:*GFP-crASNAP*.*1*;*asnap-1*/+ (C), or *UBQ10p*:*GFP-crASNAP*.*2*;*asnap-1*/+ (D). (E-L) Alexander staining of a dehiscing anther (E-H) or pollen grains (I-L) from wild-type (E, I), *ASNAPg*;*asnap-1* (F, J), *UBQ10p*:*GFP-crASNAP*.*1*;*asnap-1*/+ (G, K), or *UBQ10p*:*GFP-crASNAP*.*2*;*asnap-1*/+ (H, L) plants. (M-P) DAPI staining of mature pollen from wild-type (M), *ASNAPg*;*asnap-1* (N), *UBQ10p*:*GFP-crASNAP*.*1*;*asnap-1*/+ (O), or *UBQ10p*:*GFP-crASNAP*.*2*;*asnap-1*/+ (P) plants. DAPI channel and transmission channel images are shown at the top and the bottom, respectively. (O-P) insets are corresponding GFP channel images. (Q-S) Percentage of seed set (Q), of pollen viability by alexander staining (R), or of tricellular pollen by DAPI staining (S). Results are means ± SD (n>10) for (Q). Results are means ± SE (n = 4 involving over 300 pollen grains) for (R-S). Different letters indicate significantly different groups (One-Way ANOVA, Tukey’s multiple comparisons test, P<0.05). Bars = 1 mm for (A-D); 100 μm for (E-H); 50 μm for (I-L); 20 μm for (M-P).

Because of the male and female gametophytic lethality, the T-DNA of Cas9-ASNAP had to be retained to ensure genomic editing on *ASNAP* at the following generation. To solve this problem and also to provide more evidence that *asnap-1* was indeed a loss-of-function allele of *ASNAP*, we introduced a Cas9-resistant genomic sequence of *ASNAP* (*ASNAPg*) into *asnap-1*/+, in which the Cas9 target site was mutated without affecting the corresponding amino acids. We obtained wild-type-like plants with the *ASNAPg;asnap-1* genotype from the transformants (S4 Fig), indicating a full complementation of *asnap-1*. The *ASNAPg;asnap-1* plants expressed *crASNAP* at a level comparable to the endogenous *ASNAP* in wild type by RT-qPCRs (S4 Fig). By examining seed set (Fig 6A, 6B and 6Q) and pollen development (Fig 6F, 6J, 6N, 6R and 6S), we confirmed that the expression of *crASNAP* by introducing the genomic *ASNAP* sequence fully rescued the gametophytic lethality of *asnap-1*, further confirming the identity of *asnap-1* as the null mutant allele of *ASNAP*.

### Arabidopsis *ASNAP* encodes two functional isoforms

A close examination of the *ASNAP* genomic locus indicated that two splicing variants are encoded by *ASNAP*, both forms are constitutively expressed in various tissues and developmental stages by RT-qPCRs (S5 Fig). The second splicing form in Arabidopsis, *ASNAP*.*2*, encodes a smaller protein with an N-terminal truncation compared with ASNAP.1 (S5 Fig). Interestingly, similar N-terminal sequences were reported to mediate the interaction of yeast Sec17 or human α-SNAP with an integral membrane protein syntaxin [37], suggesting a functional distinction between ASNAP.1 and ASNAP.2.

To verify the functionality of two splicing variants, we introduced the coding sequences of *crASNAP*.*1* or *crASNAP*.*2* into *asnap-1*/+ using the constitutive promoter *UBQ10p*. We obtained homozygous *asnap-1* plants expressing *crASNAP*.*1* but not *crASNAP*.*2*, although both transgenes were expressed to a comparably high level (Figs 6Q, 6P and S6). Compared to wild type, the *UBQ10p*:*GFP-crASNAP*.*1;asnap-1* plants were defective in root and stem growth, as well as were sterile (S6 Fig), suggesting the inability of ASNAP.1 to fully rescue the defects of *asnap-1*. However, *asnap-1*/+ plants expressing either *crASNAP*.*1* or *crASNAP*.*2* were obtained. Either *crASNAP*.*1* or *crASNAP*.*2* largely, although not fully, rescued the seed set reduction of *asnap-1*/+ (Fig 6C, 6D and 6Q), indicating a partial complementation of the female gametophytic development of *asnap-1*. The defective pollen development in *asnap-1*/+ was mostly rescued by either *crASNAP*.*1* or *crASNAP*.*2* (Fig 6G, 6H, 6K, 6L and 6O–6S), indicating a partial complementation of the male gametophytic development of *asnap-1*. These results suggested that both splicing variants are functional.

**Fig 7:**
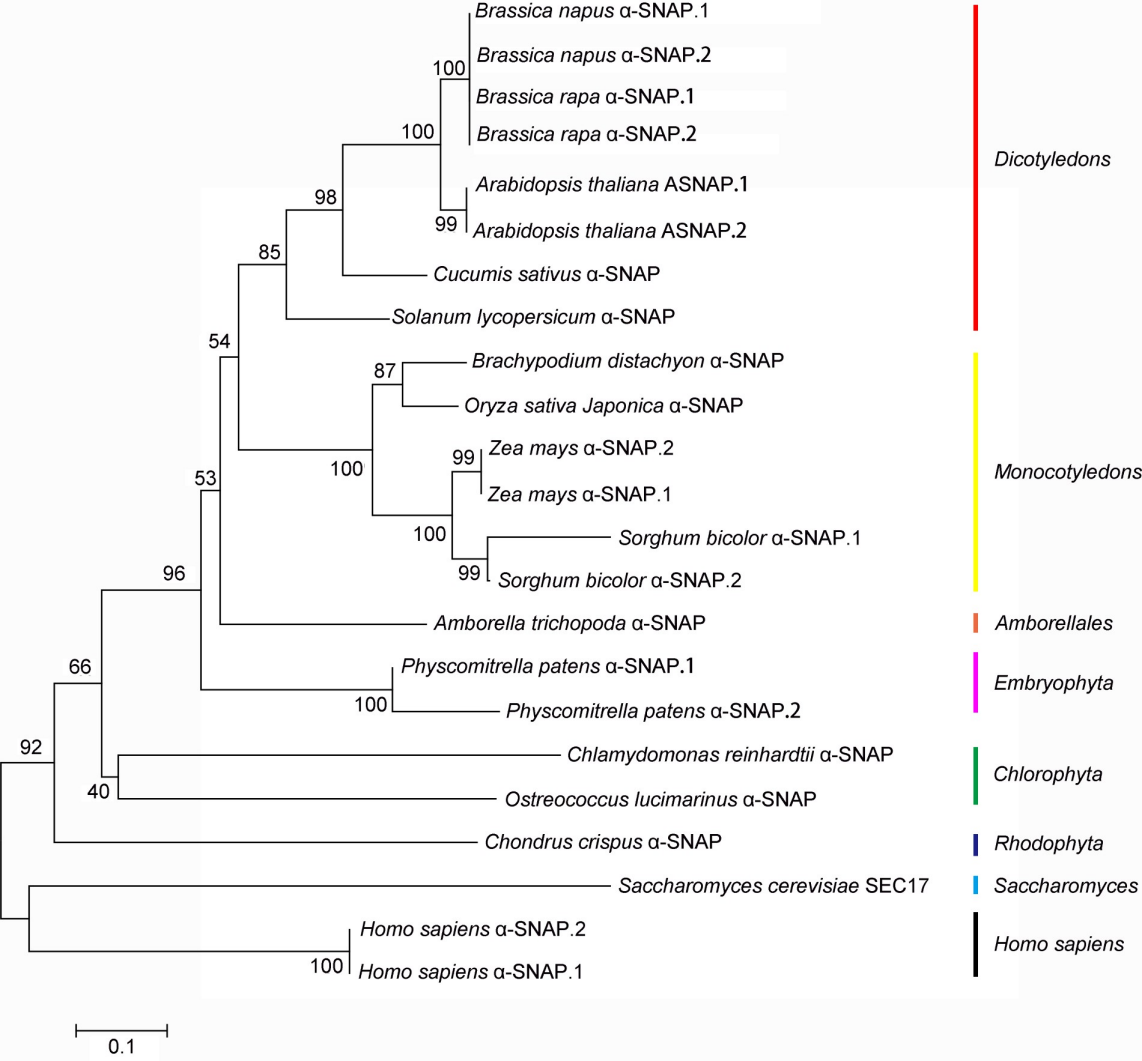
Alternative splicing of *α-SNAP* is evolutionarily recurring. Phylogenetic analysis of ASNAP orthologues using MEGA7.0. Arabidopsis protein sequences were obtained from TAIR whereas proteins from other species were obtained from the National Center for Biotechnology Information and Ensembl databases. Symbols of proteins from alternative splicing (gene symbol): NP_003818.2 and XP_011525739.1 for *Homo sapiens* α-SNAP.1 and α-SNAP.2 (8775); Pp3c18_14770V3.1 and Pp3c18_14770V3.2 for *Physcomitrella patens* α-SNAP.1 and α-SNAP.2 (Pp3c18_14770V3); XP_021307480.1 and XP_002466875.1 for *Sorghum bicolor* α-SNAP.1 and α-SNAP.2 (LOC8064551); Zm00001d033092_T001 and Zm00001d033092_T002 for *Zea mays* α-SNAP.1 and α-SNAP.2 (Zm00001d033092); XP_013742310.1 and XP_013742311.1 for *Brassica napus* α-SNAP.1 and α-SNAP.2 (LOC106445330); XP_033146543.1 and XP_009139115.1 for *Brassica rapa* α-SNAP.1 and α-SNAP.2 (LOC103863115); At3g56190.1 and At3g56190.2 for *Arabidopsis thaliana* α-SNAP.1 and α-SNAP.2 (At3g56190). Symbols of other α-SNAPs: AAA35029.1 for *Saccharomyces cerevisiae*; XP_005713623.1 for *Chondrus crispus*; OSTLU18863 for *Ostreococcus lucimarinus*; XP_001700026.1 for *Chlamydomonas reinhardtii*; AMTRs00007p00199530 for *Amborella trichopoda*; Os08g0282400 for *Oryza sativa Japonica*; BRADI_3g19810v3 for *Brachypodium distachyon*; Solyc06g050770.3 for *Solanum lycopersicum*; XP_004138403.1 for *Cucumis sativus*.

To determine whether the alternative splicing event was evolutionarily recurring, which would provide more support to its functional relevance, we searched other fully annotated plant genomes. The *α-SNAPs* in the unicellular organisms of the plant phylum, i.e. *Chondrus crispus* and *Chlamydomonas reinhardtii*, express only one isoform with all three domains comparable to yeast Sec17 (Figs 7 and S5). However, the alternative splicing of *α-SNAP* is detected in the genomes of different plant species, such as *Physcomitrella patens*, *Sorghum bicolor*, *Zea mays*, *Brassica rapa*, and *Brassica napus* (Figs 7 and S5). Interestingly, the single *α-SNAP* gene encoded in the human genome also produces two α-SNAP isoforms (Figs 7 and S5). These alternative splicing events produce two α-SNAPs with similar domain organizations as ASNAP.1 and ASNAP.2 in Arabidopsis, respectively (Figs 7 and S5). These results suggested that the alternative splicing of *α-SNAP* is an evolutionarily reoccurring event.

### Subcellular localization of two ASNAP isoforms

**Fig 8:**
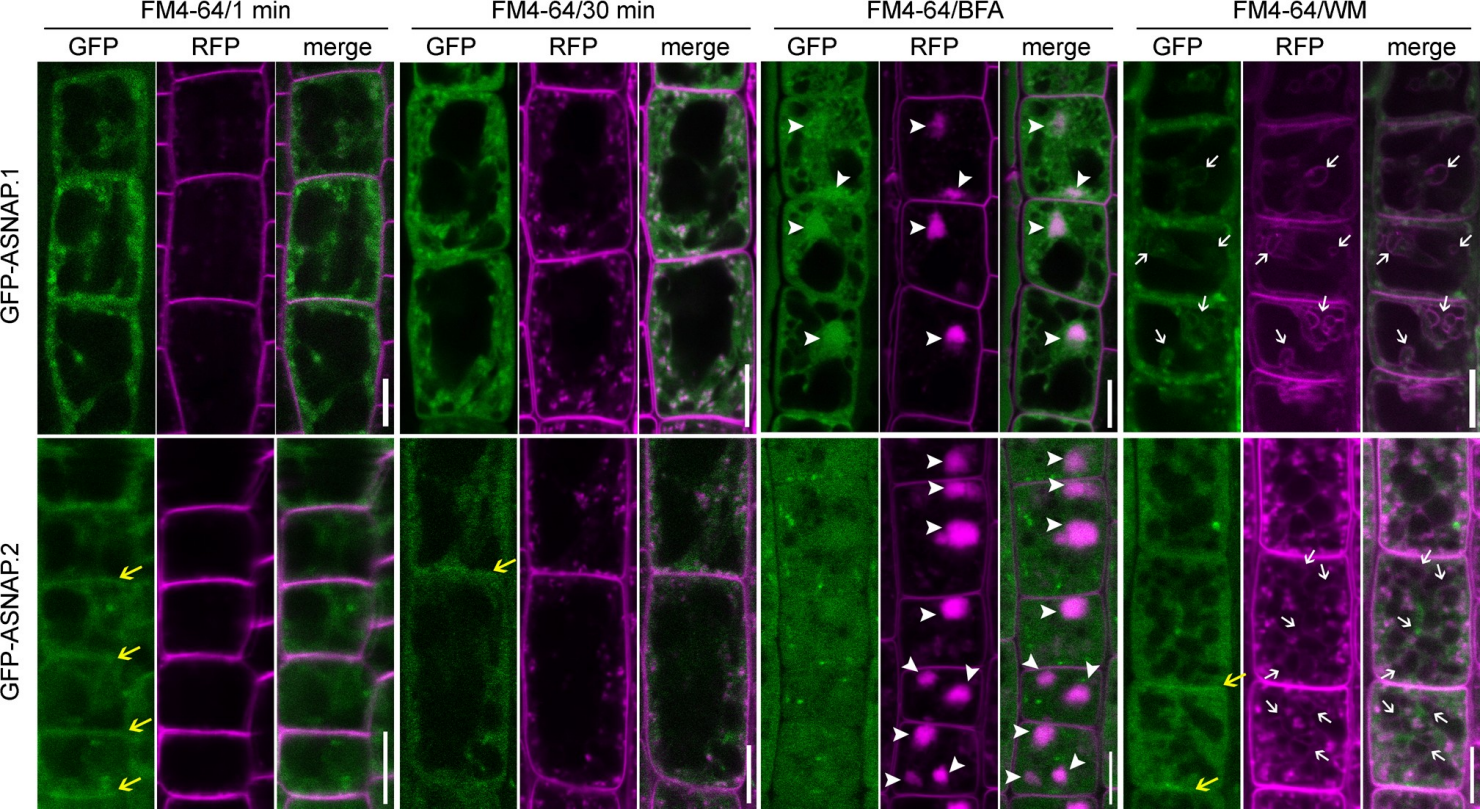
ASNAP isoforms are targeted to distinct locations. CLSM of root epidermal cells from the *UBQ10p*:GFP-ASNAP.1 or *UBQ10p*:GFP-ASNAP.2 transgenic plants. Seedlings at 4 days after germination (DAG) were pulse-labeled with 4 μM FM4-64 (FM4-64/1 min), 30 min after FM4-64 uptake (FM4-64/30 min), or FM4-64 for 5 min then treated with 50 μM BFA for 50 min (FM4-64/BFA), or FM4-64 for 5 min then treated with 33 μM WM for 30 min (FM4-64/WM). Arrowheads point at BFA compartments; white arrows point at enlarged PVC/MVB after WM treatment; yellow arrows point at PM-associated GFP signals. Bars = 10 μm.

Because Arabidopsis ASNAP.2 lacks an N-terminal sequence compared with that of Arabidopsis ASNAP.1 and yeast Sec17, we hypothesized that the two isoforms might have different subcellular localization. To test this hypothesis, examined the distribution of GFP-ASNAP.1 and GFP-ASNAP.2 by confocal laser scanning microscopy (CLSM). Root epidermal cells expressing GFP-ASNAP.1 or GFP-ASNAP.2 were pulse-labeled with the lipophilic dye FM4-64, which first indicates the plasma membrane (PM) and then is internalized to different endomembrane compartments [38]. Examination of GFP-ASNAP.1 indicated that GFP-ASNAP.1 was present in the cytoplasm as well as punctate vesicles, which partially co-localized with the internalized FM4-64 after 30 min uptake (Fig 8), indicative of the *trans*-Golgi network/early endosome (TGN/EE). Indeed, treatment of root epidermal cells with Brefeldin A (BFA), a fungal toxin that causes the accumulation of TGN/EE vesicles into so-called BFA compartments, resulted in the co-localization of GFP-ASNAP.1 and FM4-64 into BFA compartments (Fig 8), confirming that a portion of TGN/EE-associated GFP-ASNAP.1. To provide further evidence that a portion of ASNAP.1 associates with the TGN/EE, we introduced HAP13g:mRFP [39] into the *UBQ10p*:*GFP-ASNAP*.*1*-transgenic plants. CLSM of the *HAP13g*:*mRFP*;*UBQ10p*:*GFP-ASNAP*.*1* plants showed that GFP-ASNAP.1 was partially colocalized with HAP13-mRFP (S7 Fig), a marker for the TGN/EE. On the other hand, wortmannin (WM) caused the formation of FM4-64-positive rings, previously reported to be enlarged prevacuolar compartments/multivesicular bodies (PVC/MVB) [40]. These ring-shaped compartments contained also GFP-ASNAP.1 (Fig 8), indicating that a portion of ASNAP.1 associated with PVC/MVB. By examining WAVE22R;*UBQ10p*:*GFP-ASNAP*.*1* plants [41], we determined that GFP-ASNAP.1 also partially associated with the Golgi apparatus (S7 Fig). These results indicated that ASNAP.1 is present both in the cytoplasm and also at various endomembrane compartments, consistent with its canonical role in the disassembly of SNARE complexes.

By contrast, GFP-ASNAP.2 was mostly present in the cytoplasm (Fig 8). Partial colocalization of GFP-ASNAP.2 with FM4-64 at the PM was also detected (Fig 8). The PM-associated GFP signals were abolished by BFA treatment (Fig 8), likely because BFA treatment enhanced endocytosis and inhibited exocytosis. However, GFP-ASNAP.2 did not accumulate into BFA-compartments positive for the co-labeled FM4-64 (Fig 8), suggesting that GFP-ASNAP.2 is not associated with the TGN/EE. In addition, GFP-ASNAP.2 was also non-detectable at WM-induced ring-like structure (Fig 8), indicating that GFP-ASNAP.2 is not associated with PVC/MVB. The distinct localization of two ASNAP isoforms suggests their functional distinction.

### Both ASNAP isoforms interact with Arabidopsis NSF

**Fig 9:**
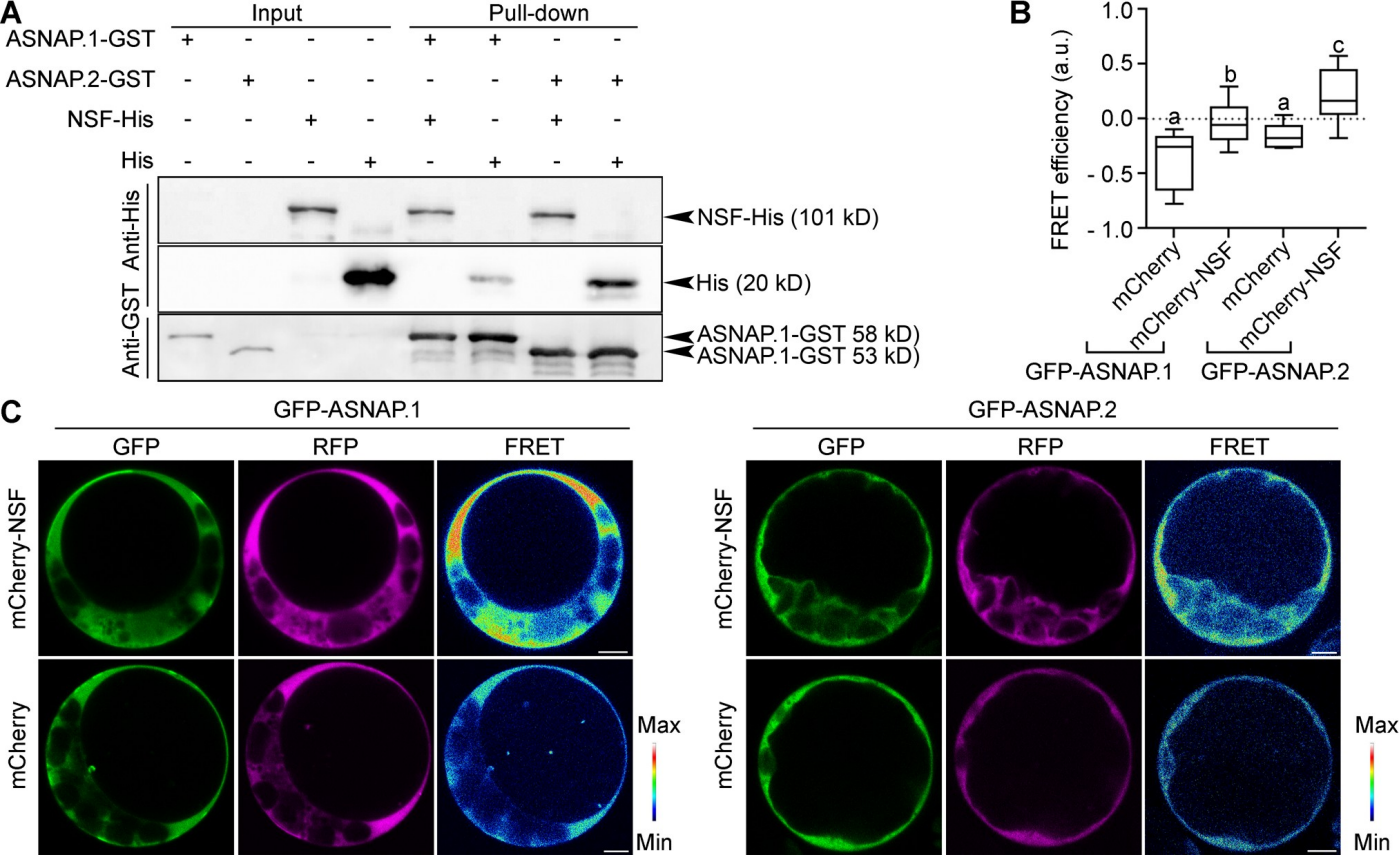
Both ASNAP isoforms interact with NSF. (A) *In vitro* pull-down assay. NSF-His was used to pull-down ASNAP.1-GST, ASNAP.2-GST. Results are representative of three biological replicates. (B-C) FRET efficiency (B) or CLSM images of FRET assays (C). FRET signals are represented in pseudo-color, covering the full range of measured values within each dataset (max to min). Results are means ± SD (n > 30). Every combination was examined with three replicate experiments. Different letters indicate significantly different groups (One-Way ANOVA, Tukey’s multiple comparisons test, P<0.05). a.u. for arbitrary unit. Bars = 5 μm.

Despite the reports on NSF-independent function of α-SNAP in mammals, the classic role of α-SNAP is to facilitate the disassembly of SNARE complex by forming a complex with NSF [25, 42]. By sequence homology, we identified a single gene in Arabidopsis encoding NSF, At4g04910, which is constitutively expressed [43] and whose coding sequence is homologous to yeast Sec18 and human NSF. Both in yeast and in mammals, Sec18/NSF interacts with Sec17/α-SNAP through its C-terminal residues [44, 45], which are conserved in both isoforms of Arabidopsis ASNAPs (S5 Fig). To determine whether Arabidopsis ASNAP interacts with NSF, we performed bimolecular fluorescence complementation (BiFC) assays. Indeed, both ASNAP.1 and ASNAP.2 showed interactions with NSF (S8 Fig). To verify that both ASNAP isoforms interact with NSF, we performed *in vitro* pull-down assays, in which his-tagged NSF was able to pull-down both ASNAP isoforms (Fig 9A). To provide further evidence for their interactions, we performed fluorescence resonance energy transfer (FRET) assays that are quantitative and allow the detection of individual interacting partners in addition to the presence of their complex. Indeed, the expression of mCherry-NSF with GFP-ASNAP.1 or GFP-ASNAP.2 showed a significant higher FRET efficiency than that of mCherry with GFP-ASNAP.1 or GFP-ASNAP.2 (Fig 9B and 9C). The interaction between ASNAPs and NSF in Arabidopsis suggests an evolutionarily conserved way of function for the SNARE-disassembly complex.

## Discussion

In this study, we demonstrated that Arabidopsis *ASNAP* is an essential gene for both male and female gametophytic development. The development of *asnap* microspores starts to show defects during PMI (Fig 3). At this stage, wild-type microspores undergo dynamic vacuolar re-organization such that a large central vacuole is fragmented into numerous small vacuoles [46]. Similarly, the development of *asnap* female gametophytes is arrested before the first mitotic division (Fig 4) when each wild-type FM produces two nuclei separated by a large central vacuole [47, 48].

Although it is still unclear whether and how vesicular dynamics affect the first mitosis during male or female gametogenesis, studies in recent years suggested a direct link between defective vacuolar dynamics and gametophytic mitosis [13–15, 23, 49, 50]. Functional loss of Arabidopsis *VACUOLELESS GAMETOPHYTES* (*VLG*) compromised vacuolar formation and fusion [49]. Its mutations resulted in defective gametophytic development at similar stages to those of *asnap* [49]. A few other mutants in which vacuolar trafficking was compromised also showed defective gametophytic development, such as the mutants of *AP-1μ*/*HAPLESS13* [13], the mutants of PI(3,5)P_2_-metabolizing enzymes [14, 15], as well as the mutants of COPII complexes [50, 51].

*ASNAP* loss-of-function could not be transmitted either through the male or the female (Table 1). The other gene in Arabidopsis whose functional loss results in the same zero male and female transmission is *YKT61* [23]. Interestingly, yeast YKT6, the homolog of Arabidopsis YKT61, plays an essential role in SNARE-complex-mediated membrane fusion, antagonistic with the SNARE-disassemble complex αSNAP/NSF [24]. It was reported that mutations at SNARE-coding genes, such as *SEC22* [18], *BET11* and *BET12* [17], *VAM3/SYP22* and *PEP12/SYP21* [19], as well as *VAMP721* and *VAMP722* [22] all compromised gametophytic development, highlighting the essential roles of fine-tuned SNARE-dynamics in ensuring plant fertility.

We demonstrated that Arabidopsis *ASNAP* encodes two isoforms (Figs 7 and S7). Although both isoforms interact with NSF (Figs 9 and S8), they may have distinct functions. By confocal imaging with fluorescence probes, we showed that ASNAP.1 associates with various endomembrane compartments, such as the TGN/EE, Golgi, PVC/MVB whereas ASNAP.2 is distributed mostly to the cytoplasm in addition to the PM (Figs 8 and S7). Introducing either ASNAP.1 or ASNAP.2 mostly restored male and female fertility of *asnap-1*/+ (Fig 6), suggesting that both isoforms are functional. In addition, ASNAP.1-transgenic plants with the homozygous *asnap-1* background grew poorly (S6 Fig), indicating that both isoforms are needed for sporophytic growth. In addition, the presence of similar alternative splicing of *α-SNAP* in human and other plant species indicates that functional distinction of two α-SNAP isoforms is evolutionarily conserved.

## Materials and methods

### Plant growth and transformation

Arabidopsis Columbia-0 ecotype was used as wild type for all experiments. Mutants including *asnap-1/+*、*asnap-2/+、asnap-3/+* were generated by CRISPR-Cas9 [35]. Plants were grown as described [52]. Stable transgenic plants were selected on half-strength MS medium supplemented with 30 μg/ml Basta salts (Sigma-Aldrich) or 25 μg/ml Hygromycin (Roche). Transgenic plants including *LAT52p*:*GUS* [53], *DD45p*:*GUS* [39], and *ES1p*:*NLS-YFP* [54, 55] were described previously.

### DNA manipulation

All constructs were generated using the Gateway technology (Invitrogen) except for CRISPR/Cas9 constructs. pENTR/D/TOPO (Invitrogen) was used to generate all entry vectors. Full-length genomic sequence of *ASNAP* was cloned by using the primer pair ZP5533/ZP5535. Then the sequence was introduced into the destination vector GW:GUS [52] to generate the expression vector ASNAPg:GUS. The full-length CDS of *ASNAP*.*1* or Cas9-resistant *ASNAP*.*1* (*crASNAP*.*1*) was cloned by using the primer pair ZP10000/ZP10001 or ZP9284/ZP9285/ZP9286/ZP9287, respectively. The full-length CDS of *ASNAP*.*2* and Cas9-resistant *ASNAP*.*2* (*crASNAP*.*2*) was cloned by using the primer pair ZP333/ZP397 or ZP9284/ZP9285/ZP9286/ZP9287, respectively. Entry vectors were used in LR reactions with the destination vector *UBQ10p*:*GFP-GW* and *35Sp*:*GFP-GW* [13, 56] to generate *UBQ10p*:*GFP-ASNAP*.*1*, *UBQ10p*:*GFP-crASNAP*.*1*, *UBQ10p*:*GFP-ASNAP*.*2*, *UBQ10p*:*GFP-crASNAP*.*2*, *35Sp*:*GFP-ASNAP*.*1*, and *35Sp*:*GFP-ASNAP*.*2*. The full-length CDS of *NSF* was cloned by using the primer pair ZP9294/ZP9295. Entry vector for *NSF* was used in LR reactions with the destination vector *35Sp*:*mCherry-GW* to generate *35Sp*:*mCherry-NSF*.

For the CRISPR/Cas9 construct used to generate the *asnap* mutants, the target site on *ASNAP* was selected using an online bioinformatics tool (http://www.genome.arizona.edu/crispr/CRISPRsearch.html) and was incorporated into forward and reverse PCR primers. The *ASNAP*-CRISPR/Cas9 cassette was generated by PCR amplifications from pCBC-DT1T2 [35] with the primers ZP5199/ZP5200/ZP5201/ZP5202. PCR products were digested with *Bsa*I and inserted into pHSE401, resulting in pHSE401-ASNAP. To verify that the CRISPR-Cas9 construct resulted in the genomic editing of *ASNAP*, the primer pair ZP5203/ZP5204 were used to amplify the genomic sequences of pHSE401-ASNAP-transformed plants. The primer ZP5203 was used to sequence the amplified genomic fragment. For the amiR-ASNAP construct, the target site and sequence-specific primers for *ASNAP* were determined using an online tool (http://wmd3.weigelworld.org/cgi-bin/webapp.cgi). The amiR-ASNAP cassette was generated by PCR amplifications from pRS300 with the primers ZP9288/ZP9289/ZP9290/ZP9291. The resultant PCR products were cloned into pENTR/D/TOPO. The entry vector was used in LR reactions with the destination vector *GPR1p*:*GW-GFP* and *UBQ10p*:*GW-GFP*.

Constructs used in BiFC assays were generated using the destination vectors pSITE-cEYFP-C1, pSITE-nEYFP-C1, pSITE-nEYFP-N1 [57]. Expression vectors used in *in vitro* pull-down assays were generated by double digestions and ligations. Coding sequences were amplified with the following primer pairs: ZP10836/ZP10837 for *NSF*, ZP10961/ZP10962 for *ASNAP*.*1*, and P732/P733 for *ASNAP*.*2*. PCR products were digested either with *Bam*HI/*Sal*I (for *NSF*) or with *Bam*HI/*Xho*I (for *ASNAP*.*1* and *ASNAP*.*2*). Digested fragments were inserted into the destination vector pET-32a [58] pre-digested with *Bam*HI/*Sal*I or *Bam*HI/*Xho*I using the pEASY-Uni Seamless Cloning and Assembly Kit (TRAN). Constructs were sequenced and analyzed using Vector NTI. All PCR amplifications were performed with Phusion hot-start high-fidelity DNA polymerase with the annealing temperature and extension times recommended by the manufacturer (Thermo Fisher Scientific). All primers are listed in S1 Table.

### RNA extraction and RT-qPCRs

Total RNAs were extracted by using a Qiagen RNeasy plant mini kit according to the manufacturer’s instructions. Oligo (dT)-primed cDNAs were synthesized by using SuperScript III reverse transcriptase with on-column DNase digestion (Invitrogen). For RT-qPCRs of *ASNAP* at diverse tissues, total RNAs were isolated from seedlings and roots at 7 DAG, from leaves at 14 DAG, from stems at 25 DAG, or from reproductive tissues at 4–5 days after anthesis. For RT-qPCRs analyzing the expression of *ASNAP* in *GPR1p*:*amiR-ASNAP*, RNAs were extracted from inflorescences. RT-qPCRs were performed with the Bio-Rad CFX96 real-time system using SYBR Green real-time PCR master mix (Toyobo) as described [52]. Primers used for RT-qPCRs are the following: ZP9086/ZP9087 for the endogenous *ASNAP*, P56/P57 for *ASNAP*.*1*, P114/P115 for *ASNAP*.*2*, and ZP12/P53 for the exogenous *ASNAP*. Primers for *GAPDH* and *ACTIN2* in RT-qPCRs were as described [52]. All primers are listed in S1 Table.

### Biochemical assays

For the purification of recombinant proteins in *in vitro* pull-down assays, GST-ASNAP.1, GST-ASNAP.2, or His-NSF were transformed into *E*. *coli* strain BL21 (Rosetta), cultured at 37°C in Lurani-Bertani medium at the presence of antibiotics (100 mg/mL ampicillin) to an OD_600_ of 0.6 to 0.8. Protein expression was induced by adding 0.8 mM isopropyl-b-D-1thiogalactopyranosid (IPTG). *In vitro* pull-down assays were performed as described [8, 39, 58]. The recombinant proteins were affinity-purified according to the manufacturer’s protocol (GE Healthcare Life Science) and analyzed by sodium dodecyl sulfate polyacrylamide gel electrophoresis (SDS-PAGE) as described [58].

### BiFC and FRET assays

BiFCs were performed in tobacco (*Nicotiana tabacum*) by transient transformations as described [58–60]. Constructs expressing mRFP-fused endomembrane marker proteins, including the tonoplast-associated INT1 [13] and PM-associated CBL1 [61] were described. For FRET assays, the vectors *35Sp*:GFP-ASNAP.1, *35Sp*:GFP-ASNAP.2, *35Sp*:mCherry-NSF and *35Sp*:mCherry were performed in Arabidopsis protoplasts by transient transformations as described [58, 62]. The calculation of FRET efficiency is as described [62].

### Phenotypic analysis

Pollen development, including Alexander staining, DAPI staining, SEM, transverse section, and TEM of developing anthers were as described [13, 14, 52, 53, 63–65]. Histochemical GUS analysis of *LAT52p*:*GUS*-pollinated pistils and aniline blue staining of pollinated pistils were performed as described [53, 65]. Methods to analyze ovule development including whole-mount ovules clearing, optical sections of developing flowers, and examination of marker-expression in embryo sacs were as described [8, 39, 55, 59].

### Fluorescence microscopy and pharmacological treatment

FM4-64 staining of root epidermal cells [13, 61, 66, 67] and Lysotracker red-staining of ovules [39, 59] were as described. Fluorescent images were captured using a Zeiss LSM 880 confocal laser scanning microscope (CLSM) with a 40/1.3 oil objective. GFP-RFP double-labeled materials were captured alternately using line-switching with the multi-track function (488-nm for GFP and 561 nm for RFP). Fluorescence was detected using a 505- to 550- nm filter for GFP or a 575- to 650-nm band-pass filter for RFP. YFP-RFP double-labeled materials were captured alternately using line-switching with the multi-track function (514 nm for YFP and 561 nm for RFP). Fluorescence was detected using a 520- to 550-nm band-pass filter for YFP or a 575- to 650-nm band-pass filter for RFP. Differential interference contrast (DIC) imaging of ovules were performed using a Zeiss Axiophot microscope with DIC optics. Image processing was performed with the Zeiss LSM image processing software (Zeiss).

### Phylogenetic analysis and genomic structure

Multiple sequence alignments were performed using the MEGA7 software package and VectorNTI. An unrooted phylogenetic tree was calculated with the neighbor-joining method, and tree topology robustness was tested by bootstrap analysis of 1,000 replicates. Alignment analysis of ASNAPs were performed by using VectorNTI software. All parameters correspond to default definitions.

### Statistical analysis

Quantification data are analyzed by using GraphPad Prism 6.02 (www.graphpad.com/scientific-software/prism/). All statistical analyses, One-Way ANOVA (Tukey’s multiple comparisons test) and *t*-test, were performed with build-in analysis tools and parameters.

### Accession numbers

Arabidopsis Genome Initiative locus identifiers for the genes mentioned in this article are: At3g56190 for *ASNAP* and At4g04910 for *NSF*.

## Supporting information

S1 Fig Reduced seed set of *asnap-1*/+ is due to female gametophytic defects. (A-B) Representative seed set of a wild-type (A) or *asnap-1*/+ pistil (B) pollinated with wild-type pollen. (C) Quantification of seed sets. Results are means ± SD (n>10). Asterisk indicates significant difference (*t*-test, P<0.05). Bars = 1 mm. Supports Fig 2.
(PDF)

S2 Fig *ASNAP* loss-of-function compromises pollen development. (A) CLSM of developing wild type or *asnap-1*/+ anthers at stage 9, stage 10, stage 11, or stage 12. T stands for tapetum. Arrowheads point at defective microspores. Asterisks indicate degenerating pollen. (B-D) Quantitative analyses of pollen development by alexander staining for pollen viability (Viability) (B), by DAPI staining for the development of tricellular pollen (Nuclei) (C), and by SEM for the rugby-shaped morphology (Morphology) (D). Results are means ± SD (n>100). Different letters indicate significant different groups (One-Way ANOVA, Tukey’s multiple comparisons test, P<0.05). Bars = 10 μm. Supports Fig 3.
(PDF)

S3 Fig Downregulating *ASNAP* constitutively compromised plant growth and fertility. (A) Relative transcript abundance of *ASNAP* (non-discriminative for splicing variants) in wild-type and two lines of the *UBQ10p*:*ami-ASNAP* seedlings (#1 and #2) at 1 week after germination (WAG). Results are means ± SE (n = 3). (B) Representative wild-type and two lines of the *UBQ10p*:*ami-ASNAP* seedlings at 1 WAG. Three seedlings of each genotype growing on the same plate are shown. (C) Primary root length at 1 WAG. Results are means ± SE (n = 9). For each biological replicate, 6 seedlings of each genotype from the same plates were examined. (D) Representative wild-type and two lines of the *UBQ10p*:*ami-ASNAP* plants at 3 WAG. (E) Representative siliques from wild type and two lines of the *UBQ10p*:*ami-ASNAP* plants. (F-K) Alexander staining of a maturing anther (F, H, J) or pollen grains (G, I, K) from wild type (F, G), line 1 (H, I), or line 2 (J, K) of the *UBQ10p*:*ami-ASNAP* plants. (L-N) DAPI staining of pollen grains released from wild type (L), line 1 (M), or line 2 (N) of the *UBQ10p*:*ami-ASNAP* plants. DAPI channel and transmission channel images are shown from top to bottom. (O) Percentage of DAPI-stained tricellular pollen from wild type and two lines of the *UBQ10p*:*ami-ASNAP* plants. Results are means ± SE (n>10). (P-R) Representative scanning electron micrographs (SEMs) of pollen (P, Q) or pollen coat structure (R) from wild type and two lines of the *UBQ10p*:*ami-ASNAP* plants. Different letters in (A, C, O) indicate significantly different groups (One-Way ANOVA, Tukey’s multiple comparisons test, P<0.05). Bars = 1 mm for (B, D, E); 100 μm for (F, H, J); 50 μm for (G, I, K, P); 20 μm for (L-N); 5 μm for (Q); 1 μm for (R). Supports Figs 1 and 5.
(PDF)

S4 Fig Functional loss of *ASNAP* is fully rescued by a Cas9-resistant ASNAP genomic fragment. (A) Relative transcript abundance of *ASNAP* (non-discriminative for splicing variants) in wild-type and *ASNAPg*;*asnap-1* seedlings at 1 WAG. Results are means ± SE (n = 3). P value (*t*-test) is shown on top of the columns. (B-D) Representative wild-type (left) or *ASNAPg*;*asnap-1* plants (right) at 1 WAG (B), 3 WAG (C), or 5 WAG (D). Supports Fig 6.
(PDF)

S5 Fig Arabidopsis *ASNAP* encodes two isoforms. (A) Quantitative real-time PCRs (RT-qPCRs) of *ASNAP* among different Arabidopsis tissues. Results shown are means ± SE (n = 3). Each biological replicate was repeated three times with similar results. (B) Sequence alignment of α-SNAPs and their splicing variants from yeast, human, and Arabidopsis. Yellow-highlighted amino acids are identical while green and blue highlighted amino acids are similar in side chains. Lilac boxes indicate predicted tetratricopeptide-repeat domain (TPR). The blue box indicates coil-coil domain (InterPro). Arabidopsis protein sequence were obtained from TAIR, whereas proteins from other species were obtained from the National Center for Biotechnology Information. Species prefixes are as follows: *Sc*, *Saccharomyces cerevisiae* (AAA35029.1); *Hs*, *Homo sapiens* (NP_003818.2 and XP_011525739.1); *At*, *Arabidopsis thaliana* (At3G56190.1 and At3G56190.2).
(PDF)

S6 Fig *UBQ10p:GFP-crASNAP.1;asnap-1* plants are defective in growth and fertility. (A) Relative transcript abundance of *ASNAP* (non-discriminative for splicing variants) in wild type versus *ASNAP*.*1* in the *UBQ10p*:*GFP-crASNAP*.*1*;*asnap-1* line. Results are means ± SE (n = 3). There is no significant difference between wild type and the complementation line (*t*-test, P>0.05). (B-C) Representative wild type or *UBQ10p*:*GFP-crASNAP*.*1*;*asnap-1* (Comp) at 1 WAG (B) or 6 WAG (C). (D-E) Representative alexander staining of a mature anther from wild type (D) or from the *UBQ10p*:*GFP-crASNAP*.*1*;*asnap-1* line (E). (F-G) A representative silique from wild type (F) or from the *UBQ10p*:*GFP-crASNAP*.*1*;*asnap-1* line (G). Out of 20 siliques examined, none from the *UBQ10p*:*GFP-crASNAP*.*1*;*asnap-1* line set seeds. Bars = 2 mm for (B); 1 cm for (C); 100 μm for (D-E); 1 mm for (F, G). Supports Figs 6 and 7.
(PDF)

S7 Fig ASNAP.1 is associated with endomembrane compartments. CLSM of root epidermal cells from *UBQ10p*:*GFP-ASNAP*.*1*;WAVE22R or *UBQ10p*:*GFP-ASNAP*.*1*;HAP13g:mRFP transgenic seedlings. RFP channels indicate either a mRFP-labeled Golgi marker (WAVE22R) or TGN/EE marker (HAP13-mRFP). Bars = 10 μm. Supports Fig 8.
(PDF)

S8 Fig Both ASNAP isoforms interact with NSF. Representative CLSM of tobacco leaf epidermal cells expressing corresponding proteins in bimolecular fluorescence complementation (BiFC) assays. For BiFC assays, agrobacterium transformed with 35S:CBL1-mRFP (as a marker for the PM) or with 35S:mRFP-INT1 (as a marker for the tonoplast) is co-infiltrated with agrobacterium transformed with YN- and YC-vectors in a 1:1 ratio and 90 RFP-positive cells for every BiFC combination from three replicates were examined for YFP signals. Numbers at the bottom indicate displayed/transformed epidermal cells. Bars = 10 μm. Supports Fig 9.
(PDF)

S1 Table Oligos used in this study. (PDF)
