# Supplementary material for: The canonical α-SNAP is essential for gametophytic development in Arabidopsis
Source: PLoS Genet. 2021 Apr 22;17(4):e1009505. doi: 10.1371/journal.pgen.1009505 (PMC8096068; doi:10.1371/journal.pgen.1009505)
Supplement: S1 Fig — (A-B) Representative seed set of a wild-type (A) or asnap-1/+ pistil (B) pollinated with wild-type pollen. (C) Quantification of seed sets. Results are means ± SD (n>10). Asterisk indicates significant difference (t-test, P<0.05). Bars = 1 mm. Supports Fig 2. (PDF) [file pgen.1009505.s001.pdf]

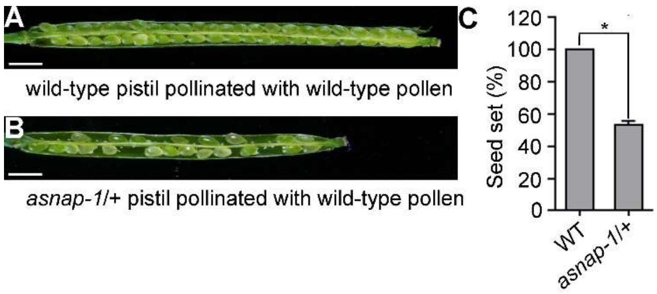

S1 Fig. Reduced seed set of *asnap-1*/+ is due to female gametophytic defects.
(A-B) Representative seed set of a wild-type (A) or *asnap-1*/+ pistil (B) pollinated with wild-type pollen. (C) Quantification of seed sets. Results are means ± SD (n>10). Asterisk indicates significant difference (*t*-test, P<0.05). Bars = 1 mm
Supports Figure 2.
