# Supplementary material for: The canonical α-SNAP is essential for gametophytic development in Arabidopsis
Source: PLoS Genet. 2021 Apr 22;17(4):e1009505. doi: 10.1371/journal.pgen.1009505 (PMC8096068; doi:10.1371/journal.pgen.1009505)
Supplement: S2 Fig — (A) CLSM of developing wild type or asnap-1/+ anthers at stage 9, stage 10, stage 11, or stage 12. T stands for tapetum. Arrowheads point at defective microspores. Asterisks indicate degenerating pollen. (B-D) Quantitative analyses of pollen development by alexander staining for pollen viability (Viability) (B), by DAPI staining for the development of tricellular pollen (Nuclei) (C), and by SEM for the rugby-shaped morphology (Morphology) (D). Results are means ± SD (n>100). Different letters indicate significant different groups (One-Way ANOVA, Tukey’s multiple comparisons test, P<0.05). Bars = 10 μm. Supports Fig 3. (PDF) [file pgen.1009505.s002.pdf]

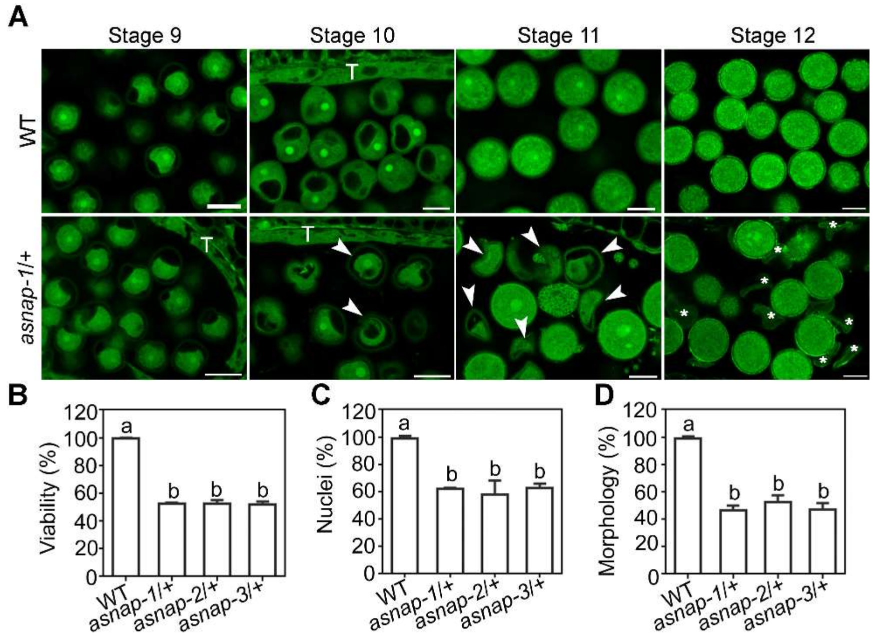

S2 Fig. *ASNAP* loss-of-function compromises pollen development.

(A) CLSM of developing wild type or *asnap-1*/+ anthers at stage 9, stage 10, stage 11, or stage 12. T stands for tapetum. Arrowheads point at defective microspores. Asterisks indicate degenerating pollen. (B-D) Quantitative analyses of pollen development by alexander staining for pollen viability (Viability) (B), by DAPI staining for the development of tricellular pollen (Nuclei) (C), and by SEM for the rugby-shaped morphology (Morphology) (D). Results are means ± SD (n>100). Different letters indicate significant different groups (One-Way ANOVA, Tukey's multiple comparisons test, P<0.05). Bars = 10 µm.

Supports Figure 3.
