# Supplementary material for: The canonical α-SNAP is essential for gametophytic development in Arabidopsis
Source: PLoS Genet. 2021 Apr 22;17(4):e1009505. doi: 10.1371/journal.pgen.1009505 (PMC8096068; doi:10.1371/journal.pgen.1009505)
Supplement: S3 Fig — (A) Relative transcript abundance of ASNAP (non-discriminative for splicing variants) in wild-type and two lines of the UBQ10p:ami-ASNAP seedlings (#1 and #2) at 1 week after germination (WAG). Results are means ± SE (n = 3). (B) Representative wild-type and two lines of the UBQ10p:ami-ASNAP seedlings at 1 WAG. Three seedlings of each genotype growing on the same plate are shown. (C) Primary root length at 1 WAG. Results are means ± SE (n = 9). For each biological replicate, 6 seedlings of each genotype from the same plates were examined. (D) Representative wild-type and two lines of the UBQ10p:ami-ASNAP plants at 3 WAG. (E) Representative siliques from wild type and two lines of the UBQ10p:ami-ASNAP plants. (F-K) Alexander staining of a maturing anther (F, H, J) or pollen grains (G, I, K) from wild type (F, G), line 1 (H, I), or line 2 (J, K) of the UBQ10p:ami-ASNAP plants. (L-N) DAPI staining of pollen grains released from wild type (L), line 1 (M), or line 2 (N) of the UBQ10p:ami-ASNAP plants. DAPI channel and transmission channel images are shown from top to bottom. (O) Percentage of DAPI-stained tricellular pollen from wild type and two lines of the UBQ10p:ami-ASNAP plants. Results are means ± SE (n>10). (P-R) Representative scanning electron micrographs (SEMs) of pollen (P, Q) or pollen coat structure (R) from wild type and two lines of the UBQ10p:ami-ASNAP plants. Different letters in (A, C, O) indicate significantly different groups (One-Way ANOVA, Tukey’s multiple comparisons test, P<0.05). Bars = 1 mm for (B, D, E); 100 μm for (F, H, J); 50 μm for (G, I, K, P); 20 μm for (L-N); 5 μm for (Q); 1 μm for (R). Supports Figs 1 and 5. (PDF) [file pgen.1009505.s003.pdf]

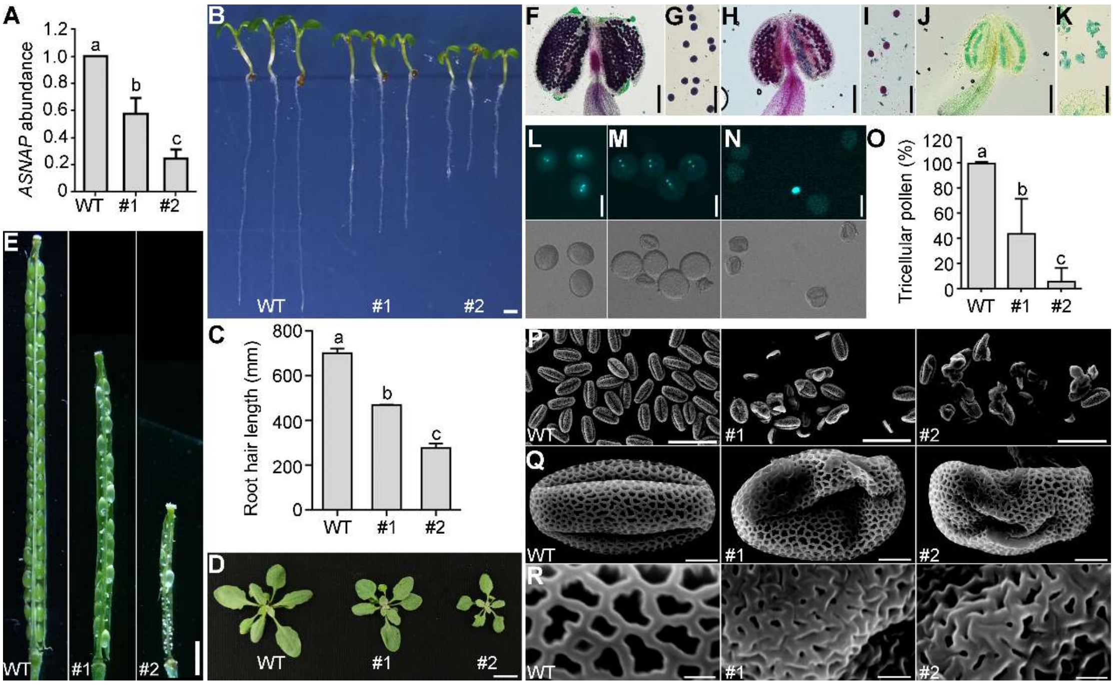

S3 Fig. Downregulating *ASNAP* constitutively compromised plant growth and fertility. (A) Relative transcript abundance of *ASNAP* (non-discriminative for splicing variants) in wild-type and two lines of the *UBQ10p:ami-ASNAP* seedlings (#1 and #2) at 1 week after germination (WAG). Results are means ± SE (n=3). (B) Representative wild-type and two lines of the *UBQ10p:ami-ASNAP* seedlings at 1 WAG. Three seedlings of each genotype growing on the same plate are shown. (C) Primary root length at 1 WAG. Results are means ± SE (n=9). For each biological replicate, 6 seedlings of each genotype from the same plates were examined. (D) Representative wild-type and two lines of the *UBQ10p:ami-ASNAP* plants at 3 WAG. (E) Representative siliques from wild type and two lines of the *UBQ10p:ami-ASNAP* plants. (F-K) Alexander staining of a maturing anther (F, H, J) or pollen grains (G, I, K) from wild type (F, G), line 1 (H, I), or line 2 (J, K) of the *UBQ10p:ami-ASNAP* plants. (L-N) DAPI staining of pollen grains released from wild type (L), line 1 (M), or line 2 (N) of the *UBQ10p:ami-ASNAP* plants. DAPI channel and transmission channel images are shown from top to bottom. (O) Percentage of DAPI-stained tricellular pollen from wild type and two lines of the *UBQ10p:ami-ASNAP* plants. Results are means ± SE (n>10). (P-R) Representative scanning electron micrographs (SEMs) of pollen (P, Q) or pollen coat structure (R) from wild type and two lines of the *UBQ10p:ami-ASNAP* plants. Different letters in (A, C, O) indicate significantly different groups (One-Way ANOVA, Tukey's multiple comparisons test, P<0.05). Bars = 1 mm for (B, D, E); 100 µm for (F, H, G); 50 µm for (G, I, K, P); 20 µm for (L-N); 5 µm for (Q); 1 µm for (R).
Supports Figure 1 and Figure 5.
