# Supplementary material for: The canonical α-SNAP is essential for gametophytic development in Arabidopsis
Source: PLoS Genet. 2021 Apr 22;17(4):e1009505. doi: 10.1371/journal.pgen.1009505 (PMC8096068; doi:10.1371/journal.pgen.1009505)
Supplement: S4 Fig — (A) Relative transcript abundance of ASNAP (non-discriminative for splicing variants) in wild-type and ASNAPg;asnap-1 seedlings at 1 WAG. Results are means ± SE (n = 3). P value (t-test) is shown on top of the columns. (B-D) Representative wild-type (left) or ASNAPg;asnap-1 plants (right) at 1 WAG (B), 3 WAG (C), or 5 WAG (D). Supports Fig 6. (PDF) [file pgen.1009505.s004.pdf]

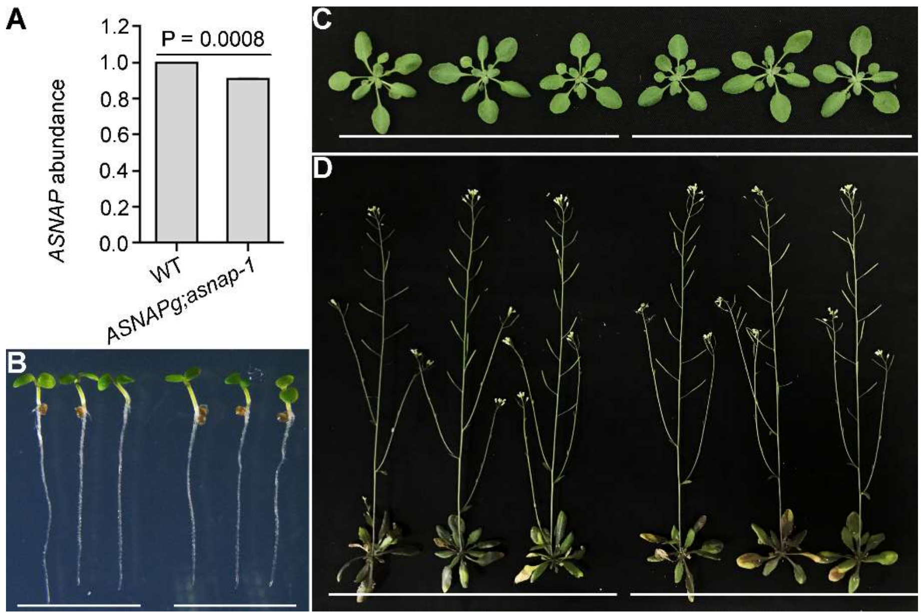

S4 Fig. Functional loss of *ASNAP* is fully rescued by a Cas9-resistant *ASNAP* genomic fragment.

(A) Relative transcript abundance of *ASNAP* (non-discriminative for splicing variants) in wild-type and *ASNAPg*;*asnap-1* seedlings at 1 WAG. Results are means ± SE (n=3). P value (*t*-test) is shown on top of the columns. (B-D) Representative wild-type (left) or *ASNAPg*;*asnap-1* plants (right) at 1 WAG (B), 3 WAG (C), or 5 WAG (D).

Supports Figure 6.
