# Supplementary material for: The canonical α-SNAP is essential for gametophytic development in Arabidopsis
Source: PLoS Genet. 2021 Apr 22;17(4):e1009505. doi: 10.1371/journal.pgen.1009505 (PMC8096068; doi:10.1371/journal.pgen.1009505)
Supplement: S5 Fig — (A) Quantitative real-time PCRs (RT-qPCRs) of ASNAP among different Arabidopsis tissues. Results shown are means ± SE (n = 3). Each biological replicate was repeated three times with similar results. (B) Sequence alignment of α-SNAPs and their splicing variants from yeast, human, and Arabidopsis. Yellow-highlighted amino acids are identical while green and blue highlighted amino acids are similar in side chains. Lilac boxes indicate predicted tetratricopeptide-repeat domain (TPR). The blue box indicates coil-coil domain (InterPro). Arabidopsis protein sequence were obtained from TAIR, whereas proteins from other species were obtained from the National Center for Biotechnology Information. Species prefixes are as follows: Sc, Saccharomyces cerevisiae (AAA35029.1); Hs, Homo sapiens (NP_003818.2 and XP_011525739.1); At, Arabidopsis thaliana (At3G56190.1 and At3G56190.2). (PDF) [file pgen.1009505.s005.pdf]

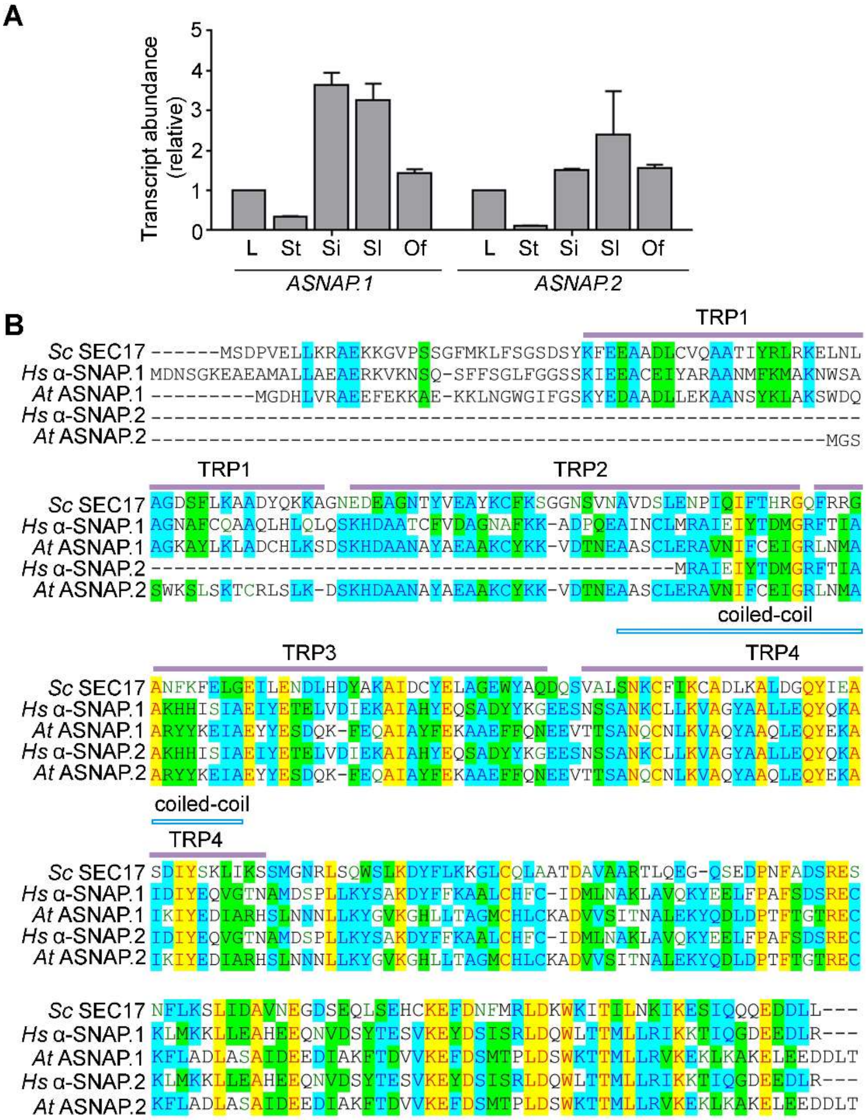

S5 Fig. Arabidopsis *ASNAP* encodes two isoforms.

(A) Quantitative real-time PCRs (RT-qPCRs) of *ASNAP* among different Arabidopsis tissues. Results shown are means ± SE (n=3). Each biological replicate was repeated three times with similar results. (B) Sequence alignment of α-SNAPs and their splicing variants from yeast, human, and Arabidopsis. Yellow-highlighted amino acids are identical while green and blue highlighted amino acids are similar in side chains. Lilac boxes indicate predicted tetratricopeptide-repeat domain (TPR). The blue box indicates coil-coil domain (InterPro). Arabidopsis protein sequence were obtained from TAIR, whereas proteins from other species were obtained from the National Center for Biotechnology Information. Species prefixes are as follows: *Sc*, *Saccharomyces cerevisiae* (AAA35029.1); *Hs*, *Homo sapiens* (NP_003818.2); *At*, *Arabidopsis thaliana* (At3G56190).
