# Supplementary material for: The canonical α-SNAP is essential for gametophytic development in Arabidopsis
Source: PLoS Genet. 2021 Apr 22;17(4):e1009505. doi: 10.1371/journal.pgen.1009505 (PMC8096068; doi:10.1371/journal.pgen.1009505)
Supplement: S6 Fig — (A) Relative transcript abundance of ASNAP (non-discriminative for splicing variants) in wild type versus ASNAP.1 in the UBQ10p:GFP-crASNAP.1;asnap-1 line. Results are means ± SE (n = 3). There is no significant difference between wild type and the complementation line (t-test, P>0.05). (B-C) Representative wild type or UBQ10p:GFP-crASNAP.1;asnap-1 (Comp) at 1 WAG (B) or 6 WAG (C). (D-E) Representative alexander staining of a mature anther from wild type (D) or from the UBQ10p:GFP-crASNAP.1;asnap-1 line (E). (F-G) A representative silique from wild type (F) or from the UBQ10p:GFP-crASNAP.1;asnap-1 line (G). Out of 20 siliques examined, none from the UBQ10p:GFP-crASNAP.1;asnap-1 line set seeds. Bars = 2 mm for (B); 1 cm for (C); 100 μm for (D-E); 1 mm for (F, G). Supports Figs 6 and 7. (PDF) [file pgen.1009505.s006.pdf]

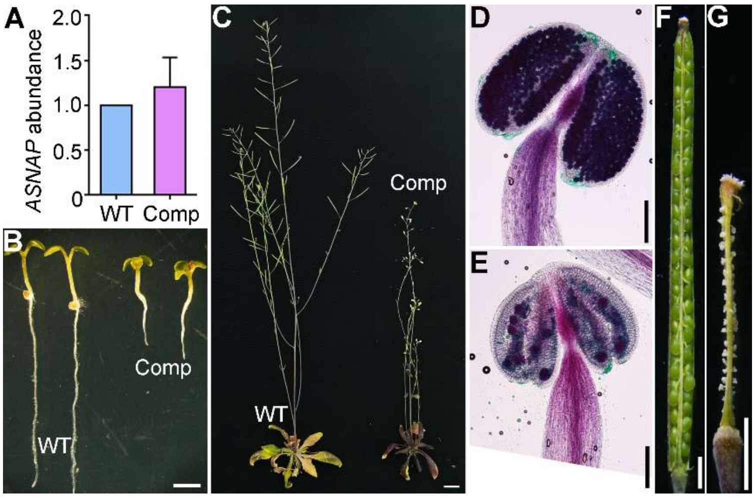

S6 Fig. *UBQ10p*:*GFP-crASNAP.1*;*asnap-1* plants are defective in growth and fertility. (A) Relative transcript abundance of *ASNAP* (non-discriminative for splicing variants) in wild type versus *ASNAP.1* in the *UBQ10p*:*GFP-crASNAP.1*;*asnap-1* line. Results are means ± SE (n=3). There is no significant difference between wild type and the complementation line (*t*-test, P>0.05). (B-C) Representative wild type or *UBQ10p*:*GFP-crASNAP.1*;*asnap-1* (Comp) at 1 WAG (B) or 6 WAG (C). (D-E) Representative alexander staining of a mature anther from wild type (D) or from the *UBQ10p*:*GFP-crASNAP.1*;*asnap-1* line (E). (F-G) A representative silique from wild type (F) or from the *UBQ10p*:*GFP-crASNAP.1*;*asnap-1* line (G). Out of 20 siliques examined, none from the *UBQ10p*:*GFP-crASNAP.1*;*asnap-1* line set seeds. Bars = 2 mm for (B); 1 cm for (C); 100 µm for (D-E); 1 mm for (F, G).

Supports Figure 6 and Figure 7.
