# Supplementary material for: The canonical α-SNAP is essential for gametophytic development in Arabidopsis
Source: PLoS Genet. 2021 Apr 22;17(4):e1009505. doi: 10.1371/journal.pgen.1009505 (PMC8096068; doi:10.1371/journal.pgen.1009505)
Supplement: S7 Fig — CLSM of root epidermal cells from UBQ10p:GFP-ASNAP.1;WAVE22R or UBQ10p:GFP-ASNAP.1;HAP13g:mRFP transgenic seedlings. RFP channels indicate either a mRFP-labeled Golgi marker (WAVE22R) or TGN/EE marker (HAP13-mRFP). Bars = 10 μm. Supports Fig 8. (PDF) [file pgen.1009505.s007.pdf]

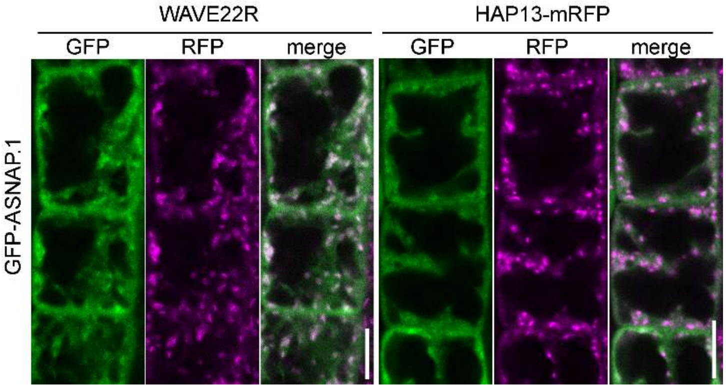

S7 Fig. ASNAP.1 is partially associated with endomembrane compartments.
CLSM of root epidermal cells from *UBQ10p:GFP-ASNAP.1*;WAVE22R or *UBQ10p:GFP-ASNAP.1*;HAP13g:mRFP transgenic seedlings. RFP channels indicate either a mRFP-labeled Golgi marker (WAVE22R) or TGN/EE marker (HAP13-mRFP). Bars = 10 µm. Supports Figure 8.
