# Supplementary material for: The canonical α-SNAP is essential for gametophytic development in Arabidopsis
Source: PLoS Genet. 2021 Apr 22;17(4):e1009505. doi: 10.1371/journal.pgen.1009505 (PMC8096068; doi:10.1371/journal.pgen.1009505)
Supplement: S8 Fig — Representative CLSM of tobacco leaf epidermal cells expressing corresponding proteins in bimolecular fluorescence complementation (BiFC) assays. For BiFC assays, agrobacterium transformed with 35S:CBL1-mRFP (as a marker for the PM) or with 35S:mRFP-INT1 (as a marker for the tonoplast) is co-infiltrated with agrobacterium transformed with YN- and YC-vectors in a 1:1 ratio and 90 RFP-positive cells for every BiFC combination from three replicates were examined for YFP signals. Numbers at the bottom indicate displayed/transformed epidermal cells. Bars = 10 μm. Supports Fig 9. (PDF) [file pgen.1009505.s008.pdf]

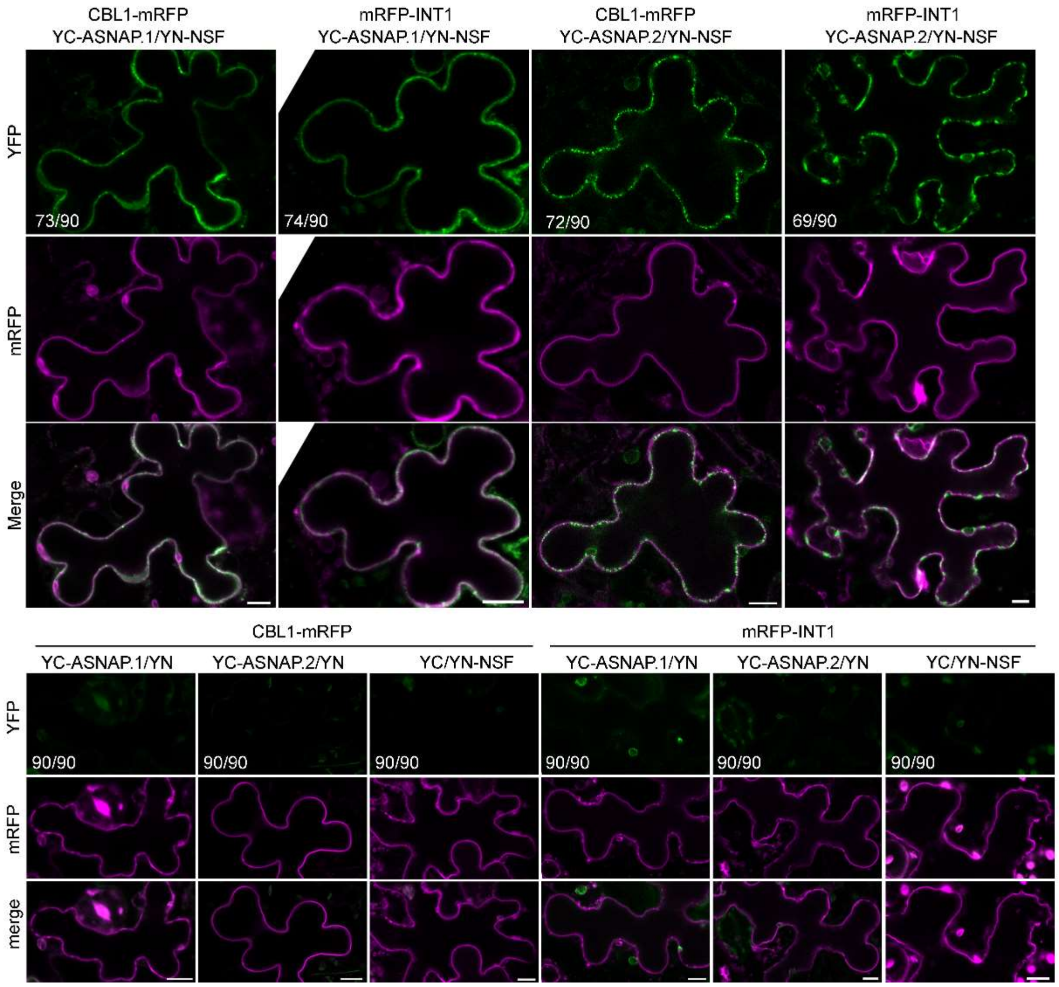

S8 Fig. Both ASNAP isoforms interact with NSF.

Representative CLSM of tobacco leaf epidermal cells expressing corresponding proteins in bimolecular fluorescence complementation (BiFC) assays. For BiFC assays, agrobacterium transformed with 35S:CBL1-mRFP (as a marker for the PM) or with 35S:mRFP-INT1 (as a marker for the tonoplast) is co-infiltrated with agrobacterium transformed with YN- and YC-vectors in a 1:1 ratio and 90 RFP-positive cells for every BiFC combination from three replicates were examined for YFP signals. Numbers at the bottom indicate displayed/transformed epidermal cells. Bars = 10 µm.

Supports Figure 9.
