# Supplementary material for: The canonical α-SNAP is essential for gametophytic development in Arabidopsis
Source: PLoS Genet. 2021 Apr 22;17(4):e1009505. doi: 10.1371/journal.pgen.1009505 (PMC8096068; doi:10.1371/journal.pgen.1009505)
Supplement: S1 Table — (PDF) [file pgen.1009505.s009.pdf]

S1 Table. Oligos used in this study.

**Application**		**No.**	**5'-3' sequences**
CRISPR/Cas9-ASNAP		ZP5199	ATATATGGTCTCGATTGGCAAACCAGTGCAATCTAAGTT
		ZP5200	TGGCAAACCAGTGCAATCTAAGTTTTAGAGCTAGAAATAGC
		ZP5201	AACCTAAGAACTTGCATTCCCGCAATCTCTTAGTCGACTCTAC
		ZP5202	ATTATTGGTCTCGAAACCTAAGAACTTGCATTCCCGCAA
ASNAP sequencing		ZP5203	GTATTTGGACTAATTTGTACATTAATGGTT
		ZP5204	CCATGATCAAGAACCAGGTAGAGTTAA
cloning	*ASNAPg*	ZP5533	CACCGTAGTTGTTATGAGGAATCGGCTTCA
		ZP5535	TGTAAGGTCATCCTCCTCTAGCTCC
	ASNAP.1	ZP10000	CACCATGGGGGATCATCTGGTGAG
		ZP10001	CTATGTAAGGTCATCCTCCTCTAGC
	ASNAP.2	P333	TTTAAGAAGGAGCCCTTCACCATGGGATCAAGCTGGAAAAGC
		P397	GGGTCGGCGCGCCCACCCTTCTATGTAAGGTCATCCTCCTCTAGC
	cr*ASNAP*	ZP9284	GCAAACCAGTGCAATCTAAAAGTTGCGCAATATGCCGCCC
		ZP9285	GGGCGGCATATTGCGCAACTTTTAGATTGCACTGGTTTGC
		ZP9286	CGGGAATGCAAGTTCTTAGCAGACCTTGCTTCTGCTATCGATG
		ZP9287	CATCGATAGCAGAAGCAAGGTCTGCTAAGAACTTGCATTCCCG
	amiR-ASNAP	ZP9288	AAGGAGCCCTTCACCGGAATATATATGTAGTATCAAATAATCGCTGAGCG
		ZP9289	TATCAAATAATCGCTGAGCGATCACAGGTCGTGATATGATTCAATTAGC
		ZP9290	TATCAAATAATCGCTGAGCGATCACAGGTCGTGATATGATTCAATTAGC
		ZP9291	TATCAAATAATCGCTGAGCGATCAAAGAGAATCAATGATCCAATTTG
	GPR1p	ZP9474	ACTTGAGCTCCAGCCAGAAATATGAAACTATCCCAGA
		ZP9475	GGACTAGTTGTTCCACAACGAACCATCTTG
	NSF	ZP9294	AAGGAGCCCTTCACCATGGCGGGTCGTTACGGATC
		ZP9295	GGCGCGCCCACCCTTCTAGCCAGTGAAGCGAATGAAGTCTCC
qRT-PCR	exogenous *ASNAP*	ZP12	AGAAGCGCGATCACATGGTC
		P53	GCGAGCTTATAGGAATTAGCTGC
	endogenous *ASNAP*	ZP9086	TGGCTGCAAGATATTACAAGGA
		ZP9087	CTTGATTGCCTTCTCATACTGC
	ASNAP.1	P56	GGTGAGAGCTGAGGAATTCG
		P57	GCGAGCTTATAGGAATTAGCTG
	ASNAP.2	P114	GTCACTTAAAGATAGCAAACATG
		P115	CTCGAACTTCTGATCAGATTC
Cloning for pull-	*ASNAP.1*	ZP10961	CTGGTTCCGCGTGGATCCATGGGGGATCATCTGGTGAG
		ZP10962	ACGATGCGGCCGCTCGAGCTATGTAAGGTCATCCTCCTCTAGC

down assays			TCC
	ASNAP.2	P732	CTGGTTCCGCGTGGATCCATGGGATCAAGCTGGAAAAG
		P733	ACGATGCGGCCGCTCGAGCTATGTAAGGTCATCCTCCTCT
	NSF	ZP10836	GCCATGGCTGATATCGGATCCATGGCGGGTCGTTACGGATC
		ZP10837	TGCGGCCGCAAGCTTGTCGACCTAGCCAGTGAAGCGAATGAAGTCTCC
